# Self-Assemblable Polymer Smart-Blocks for Temperature-Induced Injectable Hydrogel in Biomedical Applications

**DOI:** 10.3389/fchem.2020.00019

**Published:** 2020-01-31

**Authors:** Thai Thanh Hoang Thi, Le Hoang Sinh, Dai Phu Huynh, Dai Hai Nguyen, Cong Huynh

**Affiliations:** ^1^Biomaterials and Nanotechnology Research Group, Faculty of Applied Sciences, Ton Duc Thang University, Ho Chi Minh City, Vietnam; ^2^Institute of Research and Development, Duy Tan University, Da Nang, Vietnam; ^3^Faculty of Materials Technology and Polymer Research Center, Ho Chi Minh City University of Technology, VNU HCM, Ho Chi Minh City, Vietnam; ^4^Institute of Applied Materials Science, Vietnam Academy of Science and Technology, Ho Chi Minh City, Vietnam

**Keywords:** biomaterials, block copolymer, drug delivery system, hydrogels, injectable, self-assembly, temperature-sensitive, tissue engineering

## Abstract

Self-assembled temperature-induced injectable hydrogels fabricated via self-assembly of polymer smart-blocks have been widely investigated as drug delivery systems and platforms for tissue regeneration. Polymer smart-blocks that can be self-assembly play an important role in fabrication of hydrogels because they can self-assemble to induce the gelation of their copolymer in aqueous solution. The self-assembly occurs in response to an external stimulus change, such as temperature, pH, glucose, ionic strength, light, magnetic field, electric field, or their combination, which results in property transformations like hydrophobicity, ionization, and conformational change. The self-assembly smart-block based copolymers exist as a solution in aqueous media at certain conditions that are suitable for mixing with bioactive molecules and/or cells. However, this solution turns into a hydrogel due to the self-assembly of the smart-blocks under exposure to an external stimulus change *in vitro* or injection into the living body for a controllable release of loaded bioactive molecules or serving as a biomaterial scaffold for tissue regeneration. This work reports current scenery in the development of these self-assembly smart-blocks for fabrication of temperature-induced injectable physically cross-linked hydrogels and their potential application as drug delivery systems and platforms for tissue engineering.

## Introduction

Hydrogels are three-dimensional (3D) hydrophilic cross-linked polymeric networks that contain a large portion of water or biological fluids (Huynh et al., 2011a; Nguyen et al., 2015; Norouzi et al., 2016; Liu et al., 2017; Yu et al., 2018; Cirillo et al., 2019). Polymeric hydrogels have been widely used as biomaterials for drug delivery systems, cell culture platforms, wound dressing, and tissue regeneration scaffolds due to their capacity to serve as drug depots for controlled delivery of biological molecules, and their minimal cytotoxicity to the surrounding and encapsulated cells (Kim et al., 2006a, 2014; Huynh et al., 2011a; Chiang et al., 2014; Kye et al., 2014; Nguyen et al., 2015; Patel et al., 2015; Yuan et al., 2015; Norouzi et al., 2016; Wang S. J. et al., 2016; Khaliq et al., 2017; Liow et al., 2017; Liu et al., 2017; Santovena et al., 2017; Le et al., 2018; Lv et al., 2018; Yu et al., 2018; Cirillo et al., 2019). Moreover, hydrogel biomaterials offer highly porous structures and high water contain, which increases the efficiency of nutrient transportation to the encapsulated cells and facilitates the waste removal (Huynh et al., 2011a; Nguyen et al., 2015; Norouzi et al., 2016; Liu et al., 2017; Yu et al., 2018; Cirillo et al., 2019). A wide range of polymer sources, including natural-derived and synthetic polymers or their combination, has been used to fabricate hydrogels. For example, collagen, gelatin, chitosan, and are among the natural-derived polymers alginate (Sim et al., 2015; Jeon et al., 2016; Liu et al., 2017; Turabee et al., 2019). Synthetic polymer smart-blocks are synthetic polymers that can self-assembly and trigger the gelation of their copolymers under certain condition. Poly(ethylene glycol) (PEG) (Huynh et al., 2011a), poly(D,L lactic acid) (PLA) (Guo et al., 2015), poly(ε-caprolactone) (PCL) (Hyun et al., 2007), P(LA-co-glycolic acid) (PLGA) (Lee et al., 2001a), P(CL-co-LA) (PCLA) (Kang et al., 2010), P(CL-co-GA) (PCGA) (Jiang et al., 2007; Chen et al., 2016a,b), poly((R)-3-hydroxybutyrate) (PHB) (Barouti et al., 2016), poly(LA-co-δ-valerolactone) (PLVA) (Vidyasagar et al., 2017) poly(trimethylene carbonate) (PTMC) (Bat et al., 2008), poly(amino urethane) (PAU) (Dayananda et al., 2008), poly(amino ester urethane) (PAEU) (Huynh et al., 2011b) are typical examples of synthetic polymer smart-blocks, which have been widely employed for injectable hydrogel fabrication. Although natural polymer hydrogels offer many advantages, such as excellent biocompatible, biodegradable, biomimetic, low immunological stimulation, easily available, and highly versatile, many disadvantages are still remained, including low mechanical property, difficult controlling properties and/or modification degree, less-controllable degradation rate, variation between batch to batch, and disease transmission risk (Antoine et al., 2015; Jeon et al., 2015; Pina et al., 2015). On the other hand, synthetic polymer hydrogels offer uniform and designable chemical structure and properties, highly functionalability, high mechanical strength, and controllable degradation rate (Antoine et al., 2015; Pina et al., 2015).

Hydrogel networks can be crosslinked using two main strategies, chemical and physical cross-linking (Huynh et al., 2011a; Nguyen et al., 2015; Norouzi et al., 2016; Liu et al., 2017; Yu et al., 2018; Cirillo et al., 2019). Chemically cross-linked hydrogels that normally possess high mechanical strength are fabricated by employing many different chemical reactions, such as free radical, Michael-addition, Schiff base, Click, and enzymatic reactions (Nguyen et al., 2015, 2017; Jeon et al., 2016; Liu et al., 2017; Cheng et al., 2018; Huynh et al., 2018; Yu et al., 2018). However, the requirement for implantation process after fabrication and/or the need of crosslinking agents, catalysts, light, enzymes, and/or organic solvents in some cases, which may create negative effects to the encapsulated bioactive molecules and/or cells, may reduce the potential application of these systems. In opposite, physically cross-linked hydrogels can be fabricated via non-covalently linkages, such as hydrophobic interaction, hydrogen bonding and ionic interaction between the smart-blocks in polymers (Huynh et al., 2011a; Nguyen et al., 2015; Norouzi et al., 2016; Liu et al., 2017; Yu et al., 2018; Cirillo et al., 2019). Although the physically cross-linked hydrogels possess lower mechanical strength compared to chemical cross-linked hydrogels, the physically cross-linked hydrogels offer milder conditions for hydrogel formation, and the injectability fulfills a wide range of cavity geometries to form *in situ* hydrogels without the need of an operation for implantation.

Hydrogels offer many advantages for biomedical applications due to their unique properties that could be used as delivery systems to control the delivery of bioactive molecules for disease therapeutics or biomaterial scaffolds to provide structural tissue integrity in tissue regeneration (Huynh et al., 2011a; Hoffman, 2012). Hydrogels possess a high loading capacity that can be used as localized drug depots for sustained release or provide a high concentration of biological or physicochemical cues for cell activity (Li and Mooney, 2016). The release kinetics of loaded bioactive molecules can be easily tailored by controlling the hydrogel property, such as the hydrophilicity, biocompatibility, degradation and swelling rate, and crosslinking density (Slaughter et al., 2009; Hoffman, 2012; Li and Mooney, 2016). In addition, the aqueous environment in the hydrogels could help protect bioactive molecules and/or cells from damage and/or degradation while maintaining the ability to transport nutrients and waste to improve cell activity. Hydrogels can also be easily injected in deep target sites in the body and act as depots to protect the loaded bioactive molecules and cells from the immune system while maintaining good bioactivity (Slaughter et al., 2009; Huynh et al., 2011a; Hoffman, 2012; Li and Mooney, 2016). Hydrogel polymer molecules can be easily functionalized with cell targeting molecules to improve the biological interactions. For example, functionalized cell adhesive ligands, such as Arg-Gly-Asp (RGD) peptide, improved cell adhesion and spreading in dextran hydrogels (Nguyen et al., 2017). Hydrogel biomaterial scaffolds are also able to provide structural integrity to regenerated tissue constructs. Therefore, hydrogel stiffness is an important factor in regulating cell behavior, the interactions between cells, extracellular matrix, and surrounding host tissues (Vedadghavami et al., 2017). It was also reported that cells exposed to stiffer hydrogels have a higher elastic modulus in their plasma membrane and cells proliferate faster when compared to those exposed to less stiff hydrogels. However, in a stiff hydrogel, the cells migrate slower than their counterparts in softer substrates (Vedadghavami et al., 2017). Therefore, the employed hydrogels should possess a similar stiffness compared to the regenerated tissues to maximize the compatibility and integrity (Yang et al., 2009; Kim et al., 2014).

**Figure 1:**
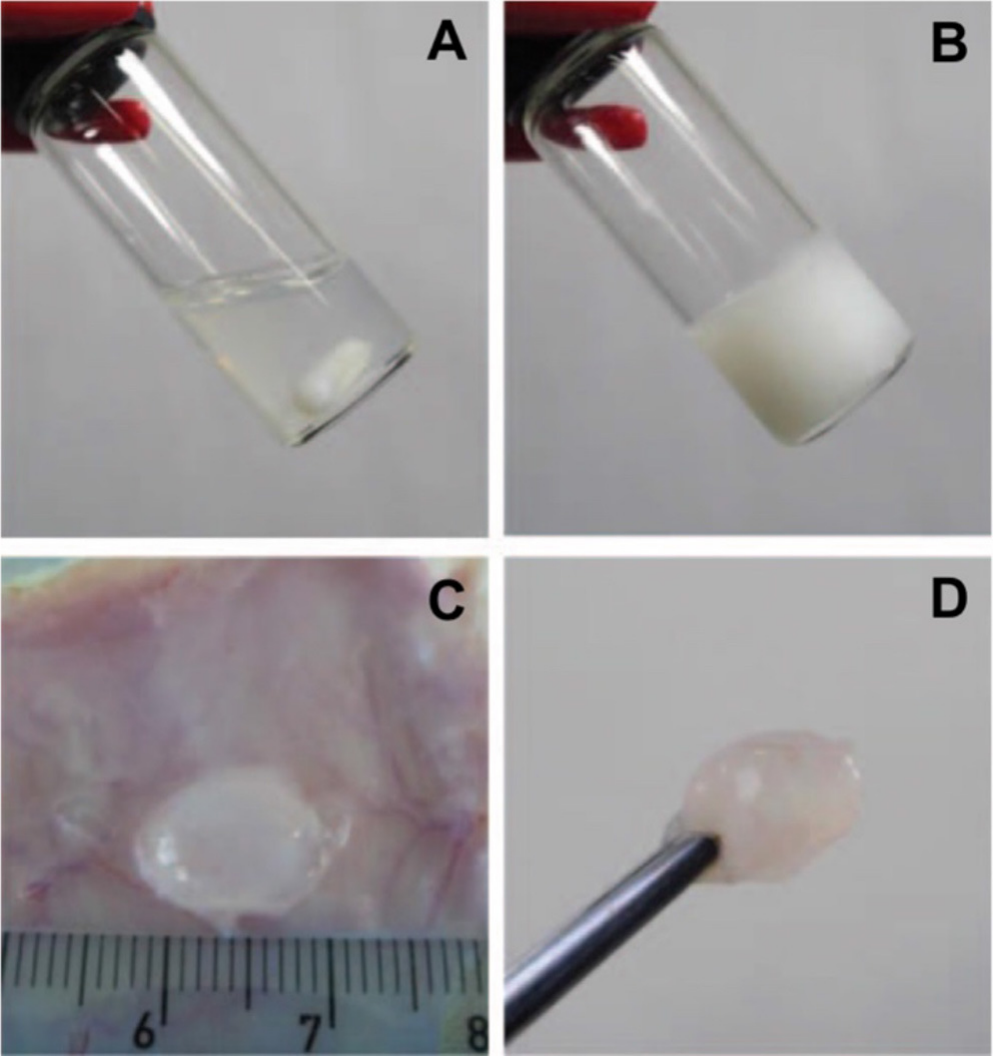
Photographs of **(A,B)** *in vitro* and **(C,D)** *in vivo* gelation of 10 wt.% PLGA-PEG-PLGA hydrogel. A polymer solution exists at 5°C **(A)** for formulation with bioactive molecules and/or cells before increasing temperature to physiological condition (**B**, 37°C) or being injected into an SD rat **(C,D)** for gel formation to serve as drug depots for sustained and localized delivery or biomaterial scaffolds for tissue regeneration. Reproduced from Loh and Oren (2012) with permission from The Royal Society of Chemistry.

Block copolymers are synthetic polymers compose at least two polymer blocks with distinct property and have been widely used for hydrogel fabrarication. Self-assembly synthetic polymer smart-blocks are segments of block copolymers that can self-assemble via property transformations, such as hydrophobicity, and ionization and conformational change, in response to the change of external environmental stimuli, including temperature, pH, glucose, ionic strength, magnetic or electric field, or their combination (Lee et al., 2001a; Hyun et al., 2007; Jiang et al., 2007; Bat et al., 2008; Dayananda et al., 2008; Kang et al., 2010; Huynh et al., 2011a,b; Yu et al., 2014a, 2018; Guo et al., 2015; Nguyen et al., 2015; Barouti et al., 2016; Chen et al., 2016a,b; Norouzi et al., 2016; Liu et al., 2017; Vidyasagar et al., 2017; Cirillo et al., 2019). These self-assembly synthetic smart-blocks play a key role in controlling the property of their copolymers in aqueous solution for fabrication of injectable self-assembled hydrogels (Huynh et al., 2011a; Nguyen et al., 2015; Norouzi et al., 2016; Yu et al., 2018; Cirillo et al., 2019). Among the stimuli signals, temperature is one of the most popular and easiest stimulus for experimentally control that has been used to induce the self-assembly of a wide range of smart-blocks and subsequently the gelation of their-derived copolymers. Injectable temperature-induced self-assembled hydrogels have been widely investigated as biomaterials for biomedical applications, especially therapeutic molecule delivery and tissue regeneration (Huynh et al., 2011a; Nguyen et al., 2015; Norouzi et al., 2016; Liu et al., 2017; Yu et al., 2018; Cirillo et al., 2019). The bioactive molecules and/or cells can be formulated with the polymer solutions prior to initiation the gelation or injection into the body without the need of an implantation procedure, to form hydrogels which serve as drug depots for sustained and localized delivery or biomaterial scaffolds for tissue regeneration (Figure 1).

This review aims to provide a development progress of self-assembly synthetic smart-blocks for fabrication of temperature-induced injectable physical cross-linked hydrogels and their potential in the delivery of therapeutic molecules and tissue regeneration. The future outlook of temperature-induced hydrogel based on these synthetic smart-blocks will also be discussed.

## Design of Self-Assembly Synthetic Smart-Blocks for Temperature-Induced Hydrogels

The physical properties of polymer smart-blocks play a critical role in fabrication of self-assembled injectable hydrogels because it regulates the ability to assemble their copolymers in response to the stimulation of temperature for forming hydrogels in aqueous solution (Huynh et al., 2011a; Nguyen et al., 2015; Norouzi et al., 2016; Yu et al., 2018; Cirillo et al., 2019). Temperature is one of the most popular signals that has been used to regulate the self-assembly of the smart-blocks to induce gelation. Temperature is also an easy stimulus to experimentally control to induce the self-assembled gelation of a wide range of smart-blocks both *in vitro* and *in vivo*. Temperature-responsive smart-blocks are amphiphilic blocks that can exhibit hydrophilic-hydrophobic transformation or conformational change in response to a temperature difference, which creates a change in physical state of an aqueous copolymer solution, e.g., a solution (sol) to a gel (Huynh et al., 2011a; Nguyen et al., 2015; Norouzi et al., 2016; Yu et al., 2018; Cirillo et al., 2019). For example, in aqueous solution, the polymers exist as a sol state at certain low temperature ranges that facilitates the incorporation of bioactive molecules and/or cells before transforming into a 3D hydrogel network (gel state) at physiological temperature (37°C) for serving as a drug depot for control the delivery and/or a biomaterial scaffold for cell growth.

**Figure 2:**
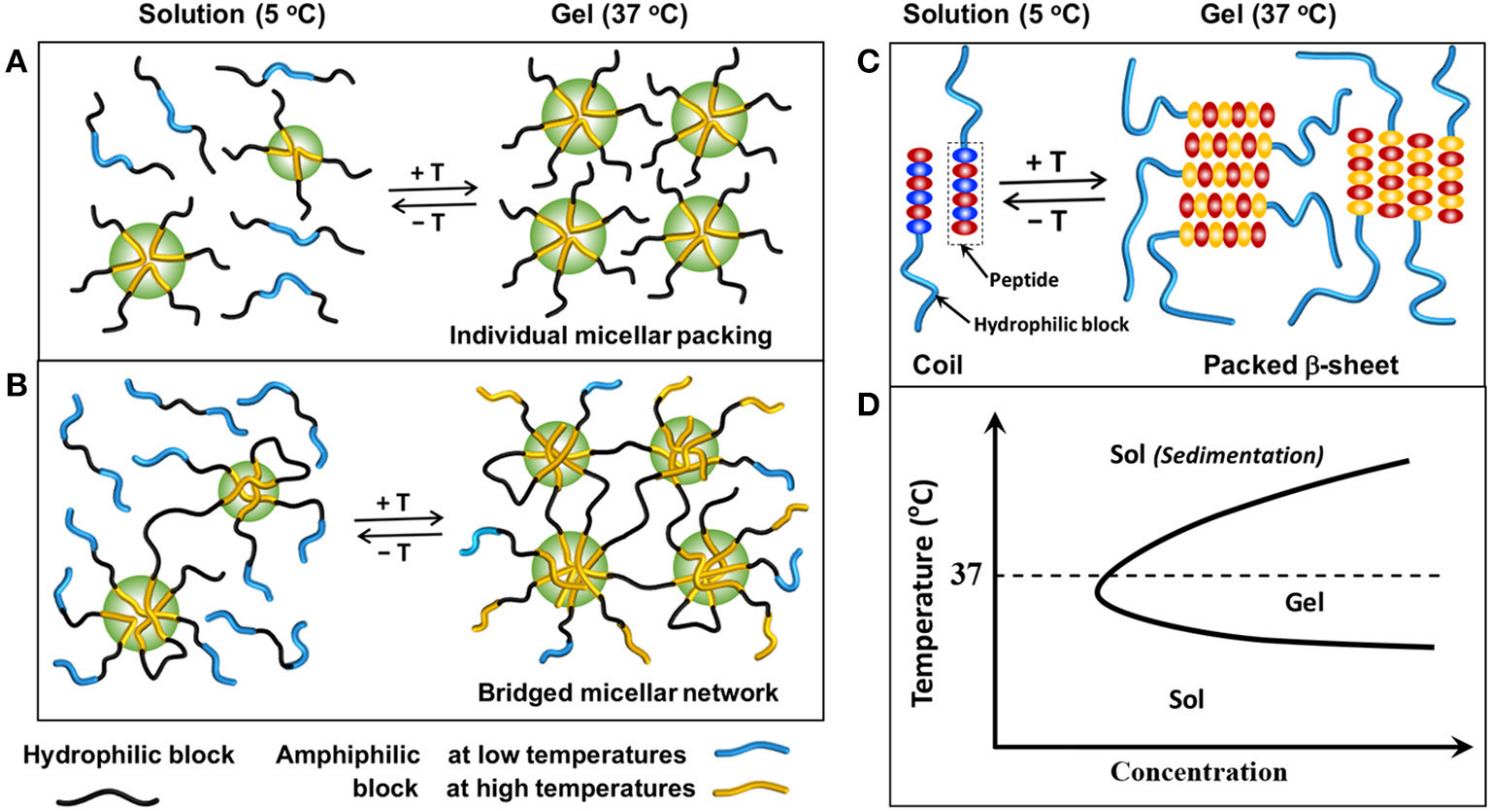
Schematic showing the sol-gel phase transition mechanisms in aqueous solution: **(A)** association of individual micelles from copolymers contain single amphiphilic smart-blocks, **(B)** association of inter-molecular micelles from copolymers contain multiple amphiphilic smart-blocks, and **(C)** conformational changes of peptide-based smart-blocks from random coils to packed β-sheets structure to form hydrogels. **(D)** A typical sol-gel phase transition of temperature-induced hydrogels, in which a polymer solution exists at low temperatures and exhibits a sol-to-gel phase transition to form a hydrogel at elevated temperatures.

There are two main mechanisms for self-assembly and subsequently gelation of temperature-sensitive smart-blocks based injectable copolymer hydrogels, including micellization and conformational transitions (Huynh et al., 2011a; Nguyen et al., 2015; Zhang et al., 2015a; Norouzi et al., 2016; Yu et al., 2018; Cirillo et al., 2019). In micellization mechanism, amphiphilic smart-blocks increase their hydrophobicity in response to temperature change, and the micellization occurs due to the hydrophobic interaction between smart-blocks. The degree of micellization can be further triggered with extended temperature change that leads to the association of individual micelles (Figure 2A) or inter-molecular micelles (Figure 2B) in polymers containing single or multiple smart-blocks in the molecules, respectively, which finally leads to the formation of 3D hydrogel networks. At low concentration, amphiphilic polymers can dissolve as individual molecules in an aqueous solution. However, polymer molecules start to interact with each other to form polymeric micelles at a specific increased polymer concentration which is defined as critical micelle concentration (CMC). The higher hydrophobicity blocks possess lower CMC values, and therefore tend to form stronger interaction micelles to trigger the gelation at lower concentration. The conformational transition gelation mechanism normally happens in peptide-based smart-blocks, which undergo transitions from random coils to packed β-sheets, and potentially further to nanofibers to form 3D hydrogel networks in response to the temperature change (Figure 2C). In either mechanism, the sequence of the smart-blocks and topology of the copolymer molecules play a critical role in controlling the gelability and gelation mechanism of the designed hydrogels which can be used as effective tools to design a suitable hydrogel system.

**Figure 3:**
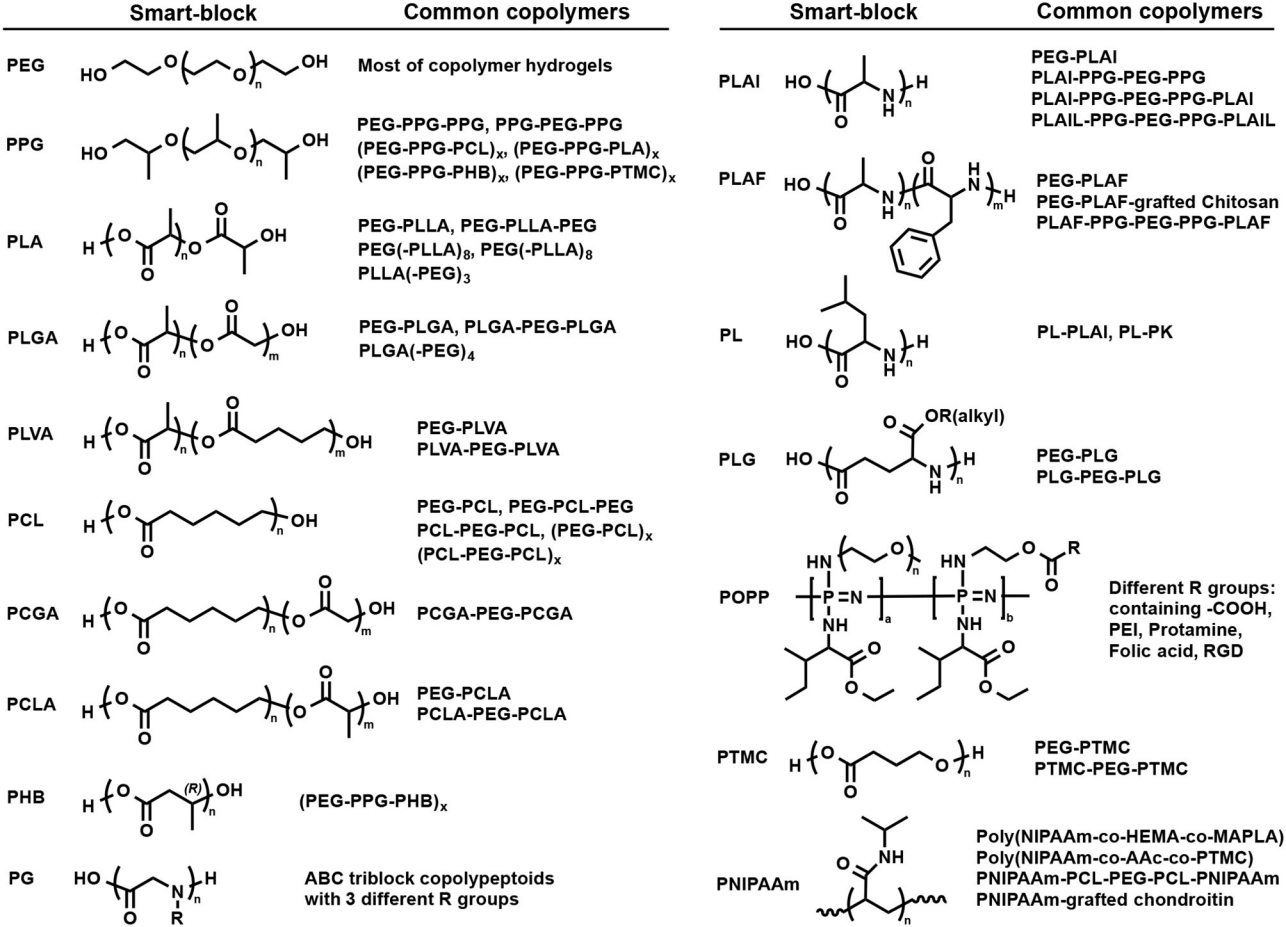
Structure of some typical self-assembly smart-blocks for fabrication of temperature-induced self-assembled injectable hydrogels.

A wide range of synthetic polymer smart-blocks exhibit a response to the temperature in an aqueous solution. However, only some of them could trigger the gelation of their (co)polymers in aqueous media at surrounding body conditions that showed potential in biomedical engineering applications. Some typical examples of temperature-sensitive gelable smart-blocks are poly(N-isopropylacrylamide) (PNIPAAm), polyethers, e.g., PEG or PEO and poly(propylene oxide) (PPG or PPO), aliphatic polyesters, aliphatic polycarbonates, and polypeptides (Figure 3). The gelation mechanism of these temperature-sensitive smart-blocks based injectable hydrogels and their potential applications in drug delivery, therapeutic treatment and tissue engineering have been widely investigated. This section discusses about the synthesis and gelation of these smart-blocks and their temperature-induced injectable hydrogels.

### Polyether Smart-Blocks

PEG (Figure 3) is a hydrophilic and neutral charge synthetic polymer with a wide range of molecular weight that has been the most popular smart-block for hydrogel preparation in biomedical applications (Huynh et al., 2011a; Nguyen et al., 2015; Norouzi et al., 2016; Yu et al., 2018; Cirillo et al., 2019). Although PEG is hydrophilic and could not trigger the gelation, it can function as a bridge to induce the cross-linking density of the hydrogels. PEG has been widely used to fabricate chemically cross-linked hydrogels for a wide range of applications, such as drug delivery and tissue regeneration (Nguyen and West, 2002; Nguyen et al., 2018). In addition, the dehydration of PEG at high temperatures reported to result in a gel-to-sol phase transition (Huynh et al., 2010). PEG is the most important smart-block for conjugation with other smart-blocks to fabricate the hydrogels that are present in most of the synthetic block copolymer hydrogels.

PPG (Figure 3) exhibits amphiphilic property at low range molecular weight or when copolymerized with other hydrophilic polymers, such as PEG, and can self-assemble to form micelles in an aqueous solution (Glatter et al., 1994; Allcock et al., 2006; Loh et al., 2010; Choi et al., 2011a; Khaliq et al., 2017; Liow et al., 2017). Its copolymer with PEG in the form of PEG-PPG-PEG copolymers, known as Pluronic (BASF) or the Synperonic PE/F (Croda) or Poloxamer (ICI), are soluble in water at low temperature with a low degree of micellization due to the self-assemble properties of PPG (Huynh et al., 2011a; Khaliq et al., 2017). By increasing temperature, the degree of micellization increases which leads to the formation of associated micellar structure and subsequently results in a sol-to-gel phase transition (Mortensen and Brown, 1993; Glatter et al., 1994; Khaliq et al., 2017; Liow et al., 2017) (Figure 2A). However, when the temperature was further elevated, the dehydration of PEG blocks lead to the reduction in micellar interactions and subsequent the collapse of the 3D hydrogel network, indicated by a gel-to-sol phase transition. Pluronic hydrogels are normally non-biodegradable, short persistent, high permeability, and low mechanical strengths that may limit their potential application (Huynh et al., 2011a; Nguyen et al., 2015; Norouzi et al., 2016; Yu et al., 2018; Cirillo et al., 2019). Scientists have modified Pluronic to adjust its critical gel concentration and sol-to-gel phase transition temperature, and improve their biodegradability, thermal and mechanical properties, and their biocompatibility (Cohn et al., 2006; Choi et al., 2011a; Loh et al., 2014; Dou et al., 2016; Wu et al., 2016a). For example, conjugating polyhedral oligosilsesquioxane (POSS) to both end of PEG-PPG-PEG copolymers via atom transfer radical polymerization (ATRP) to form POSS-PEG-PPG-PEG-POSS copolymer hydrogel could increase the sol-to-gel phase transition temperature from 22 to 33°C without changing mechanical property of the formed hydrogels (Dou et al., 2016). In addition, PPG have also been grafted (Kim D. H. et al., 2010; Nguyen et al., 2016) into or simply mixed (Jung et al., 2017) with natural polymers, such as chitosan, heparin (HEP), hyaluronic acid (HA), and gelatin, to fabricate self-assemble temperature-induced injectable hydrogels. These inherit the biocompatibility of their natural parent polymers and the temperature responsive property of PPG, for improving hydrogel properties. For example, the addition of high molecular weight HA into PEG-PPG-PEG hydrogel could enhance the gel strength due to the co-association of HA into the intermolecular micellar packing in the hydrogel structure (Jung et al., 2017).

### Aliphatic Polyester Biodegradable Smart-Blocks

Similar to PPG, amphiphilic aliphatic biodegradble polyester smart-blocks (Figure 3), including PLLA (Jeong et al., 1997, 1999a; Park et al., 2001; Hiemstra et al., 2006, 2007; Nagahama et al., 2008), PDLA (Hiemstra et al., 2006, 2007), PLA (Jeong et al., 1999a; Li et al., 2007; Chen et al., 2010; Guo et al., 2015; Shi et al., 2016), PCL (Lee et al., 2001b; Bae et al., 2006; Kim et al., 2006a; Hyun et al., 2007; Gong et al., 2009a,b; Kang et al., 2010; Peng C. L. et al., 2013), PLGA (Zhang et al., 2008; Peng S. et al., 2013; Chiang et al., 2014; Yu et al., 2014a; Yuan et al., 2015; Zhang L. et al., 2015; Wang S. J. et al., 2016; Lei et al., 2017; Santovena et al., 2017; Shen et al., 2017), PCLA (Xun et al., 2009; Kang et al., 2010; Petit et al., 2013, 2014, 2015; Sandker et al., 2013; Tellegen et al., 2018), PCGA (Jiang et al., 2007; Yu et al., 2014a; Chen et al., 2016a,b), PLVA (Peng S. et al., 2013; Vidyasagar et al., 2017), and PHB (Barouti et al., 2016; Wu et al., 2016b; Wee et al., 2017), in their copolymers with PEG can self-assemble into individual micelles or inter-molecular micelles, and then the association of individual micelles or inter-molecular micelles could induce the gelation of their copolymers (Figures 2A,B). These blocks can be easily synthesized via ring opening polymerization (ROP) technique using hydroxyl terminated molecules, such as hydroxyl-terminated PEGs, and appropriate cyclic ester derived monomers (Jeong et al., 1997). The prepared blocks can be further conjugated via urethane coupling (Jeong et al., 1997; Gong et al., 2009a; Gou et al., 2010; Hwang et al., 2010) and esterification (Li et al., 2007; Zhang et al., 2017) to achieve designated polymer structures or increase the length of the smart-blocks. They have been copolymerized with PEG to fabricate a wide range of self-assembled, biodegradable polymeric injectable hydrogels, which show potential application in drug delivery, therapeutic treatment, and tissue regeneration. This section discusses in more detail the gelation mechanisms and important factors to control the self-assembled gelation of some typical amphiphilic aliphatic polyester smart-blocks.

The first study reported the preparation of biodegradable aliphatic polyester as self-assembled polymeric injectable hydrogels for drug delivery was published in 1997 (Jeong et al., 1997). Copolymers of PLLA and PEG in the forms of PEG-PLLA diblock or PEG-PLLA-PEG triblock copolymers were synthesized via ROP, followed by urethane coupling to fabricate triblock. The gelation behavior and potential applications as drug delivery systems were investigated. In aqueous media, both PEG-PLLA and PEG-PLLA-PEG existed in the sol state at high temperatures and exhibited a sol-to-gel phase transition upon lowering down the temperature. The release of loaded-dextran, a model drug, was sustained for more than 2 weeks. Although the transition temperature can be regulated by tailoring the block length of biodegradable PLLA, hydrophobic/hydrophilic ratios, polymer end-groups, and/or stereoregularity of the PLLA (Jeong et al., 1997; Park et al., 2001; Li et al., 2007; Nagahama et al., 2008), the high temperature sol state of these hydrogels limited their application due to the potential damage of formulated bioactive molecules and/or encapsulated cells, and patients' inconvenience.

Soon after, other biodegradable aliphatic polyesters, e.g., PLGA, PCL, or PCLA, were added as self-assembly smart-blocks for temperature-induced injectable hydrogels. When the PLLA smart-block were replaced by PLGA or PCL or PCLA to generate PEG-PLGA (Zhang et al., 2008; Peng S. et al., 2013; Chiang et al., 2014; Lei et al., 2017; Shen et al., 2017), PEG-PCLA (Kang et al., 2010), and PEG-PCL (Kim et al., 2006a; Hyun et al., 2007; Kim M. H. et al., 2010; Lee et al., 2010; Kang et al., 2011) diblock or PEG-PLGA-PEG (Jeong et al., 1999b, 2000; Li, 2003; Luan et al., 2017) and PEG-PCL-PEG triblock copolymers (Bae et al., 2005; Gong et al., 2009a,b; Gou et al., 2010; Hwang et al., 2010; Yin et al., 2010; Mishra et al., 2011; Zhang et al., 2017), these copolymers existed as a sol state in aqueous media at low temperatures and exhibited a sol-gel-sol phase transition upon increasing the temperature (Figure 2D). These sol-to-gel phase transition are attributed to the micellization of hydrophobic smart-blocks, and the closed packing of individual micelles triggered by increasing temperature (Figure 2A). In addition, increasing the number of PLA smart-blocks in a single copolymer molecule, such as using two PLA blocks (e.g., PLA-PEG-PLA) (Guo et al., 2015; Shi et al., 2016), also offered a sol state window at low temperatures. The window of sol state at low temperatures offers mild conditions for formulation of bioactive molecules and/or cells before inducing the gelation or being injected into the body.

Topology of copolymers is one of the key factors that regulates the self-assembled gelation of the injectable hydrogels. Hydrogels consist of single amphiphilic aliphatic polyester smart-block in the backbone normally require higher polymer concentration to induce the gelation and the resulting hydrogels possess low mechanical properties due to their associated individual micelles triggered gelation mechanism (Kim M. H. et al., 2010; Lee et al., 2010; Kang et al., 2011; Lei et al., 2017; Luan et al., 2017; Shen et al., 2017). In contrast, copolymers consist of multiple smart-blocks in their backbone, such as PLGA-PEG-PLGA, PCGA-PEG-PCGA, PCL-PEG-PCL, PCLA-PEG-PCLA, poly(CL-co-p-dioxanone) (PCLDO)-PEG-PCLDO, and (PEG-PPG-PHB)_x_ can form hydrogels at lower polymer concentrations with higher gel strengths due to the formation of associated inter-molecular micelles (Figure 2B; Bae et al., 2005; Loh and Oren, 2012; Li et al., 2013; Yu et al., 2014a,b; Yuan et al., 2015; Zhang L. et al., 2015; Cao et al., 2016; Wang S. J. et al., 2016; Zhang et al., 2016; Santovena et al., 2017; Wee et al., 2017). For example, 25% aqueous solutions of PEG-PLGA (Luan et al., 2017) and PLGA-PEG-PLGA (Li et al., 2013; Yu et al., 2014a,b; Yuan et al., 2015) exhibited a similar reversible sol-to-gel phase transition with increasing temperature but possessed different maximum storage moduli of ~200 and 1,000 Pa, respectively. In aqueous solution, these multiple smart-blocks contained copolymers form bridged micelles at low temperatures and transform to the association of bridged micelles at higher temperatures due to the increase in hydrophobicity of the smart-blocks (Lee et al., 2001a; Shim et al., 2002; Huynh et al., 2011a; Nguyen et al., 2015; Norouzi et al., 2016; Yu et al., 2018; Cirillo et al., 2019). The linkages between micelles in these hydrogels act as additional bond to enhance the hydrogel mechanical properties compared to those that contain single smart-block (Figure 2B).

The natural hydrophobicity and crystallization of aliphatic polyester smart-blocks have a strong effect on the self-assemblability and amphiphilicity of their copolymers. For example, by increasing the hydrophobicity of original monomers in smart-blocks from PGA to PDO to PLA to PVA, and to PCL, significantly increase in hydrogel strength (Kang et al., 2010; Yu et al., 2014a; Chen et al., 2016b) and retention time of the hydrogels (Kang et al., 2010; Peng S. et al., 2013) or lower critical gel concentration (Jeong et al., 1999a; Chen et al., 2010, 2016a; Peng S. et al., 2013; Yu et al., 2014a) were observed due the decrease of CMC value. In addition, increasing the crystallization degree of polyester block leads to increasing its hydrophobicity that regulates the micellization of its copolymers at lower concentration and gelation at lower temperatures (Kim et al., 2006b; Gong et al., 2009b; Shi et al., 2016). The hydrophobicity of a combined smart-blocks can be regulated by changing their fraction. For example, increasing the PCL/PLA (Kang et al., 2010; Petit et al., 2013) and PLA/PGA (Yu et al., 2007) ratio could increase the hydrophobicity of PCLA and PLGA smart-blocks, respectively. Higher hydrophobicity blocks tend to form stronger micelle interactions at lower concentrations that lead to the formation of stronger hydrogels or hydrogel formation at lower polymer concentration. Moreover, increasing the length of smart-blocks also lead to the formation of stronger gels or hydrogels at lower polymer concentration (Chen et al., 2010; Shi et al., 2016; Zhang et al., 2017). Controlling the smart-block hydrophobicity is a flexible and useful tool in the design and fabrication of desired hydrogel systems for applications with specific requirements.

Amphiphilic aliphatic polyester smart-blocks normally contain hydroxyl end-groups that can be easily functionalized to regulate their self-assembly behavior. The gel window was significantly changed after acyl (Yu et al., 2006, 2007; Petit et al., 2013; Sandker et al., 2013) or carboxylic acid (Chang et al., 2009; Oborna et al., 2016; Rao et al., 2018) groups or a short peptide (Xun et al., 2009) were conjugated to the end-groups of polyester smart-block in PLGA-PEG-PLGA (Yu et al., 2006, 2007; Chang et al., 2009; Oborna et al., 2016) or PCLA-PEG-PCLA (Xun et al., 2009; Petit et al., 2013; Sandker et al., 2013) or PCLDO-PEG-PCLDO (Rao et al., 2018) triblock copolymers hydrogels. For example, increasing the hydrophobicity of conjugated acyl end-groups (original hydroxyl to ethanol to propanoyl) in PLGA-PEG-PLGA (Yu et al., 2006, 2007) or PCLA-PEG-PCLA (Petit et al., 2013; Sandker et al., 2013), the hydrogel can be achieved at lower temperature and/or lower polymer concentration. However, further increasing hydrophobicity of the conjugated acyl end-groups to butanonyl led to the insoluble copolymers (Yu et al., 2006). On the other hand, the gelation temperature of PCLA-PEG-PCLA (Xun et al., 2009) decreased when a short functional KRGDKK (Lys-Arg-Gly-Asp-Lys-Lys) peptide was conjugated to both ends. Cholesterol-capped PEG(PLLA)_8_ exhibited sol-gel phase transition upon increasing the temperature while the parent PEG(PLLA)_8_ could not form hydrogel in aqueous solution (Nagahama et al., 2008).

Many amphiphilic aliphatic polyester smart-blocks, such as PHB (Barouti et al., 2016; Wu et al., 2016b; Wee et al., 2017), PLA (Wu et al., 2016a), and PCL (Zheng et al., 2017), have been combined with PEG and PPG via urethane coupling to fabricate multiple smart-blocks contain temperature-induced biodegradable polymer hydrogels with much lower critical gel concentration. Tailoring the hydrophobicity or ratio between smart-blocks could alter the gel region. For example, replacing PLA (Wu et al., 2016a) in (PEG-PPG-PLA)_x_ multiblock copolymer hydrogel by more hydrophobic PBH (Wu et al., 2016b) or PCL (Zheng et al., 2017) blocks led to a significant reduction of critical gelation concentration from ~10 to 4% (w/w). Increasing the content of hydrophobic block PPG and/or PHB or replacing the PHB with poly(4-hydroxybutyrate), a more hydrophobic block, also decreased the critical gelation concentration (Wee et al., 2017). In addition, several polyester smart-blocks have been grafted into natural polymers, such as HEP (Sim et al., 2015) and gelatin (Turabee et al., 2019), and proteins, e.g., BSA (Giang Phan et al., 2019), to improve the property of fabricated hydrogels.

### Polypeptide Smart-Blocks

Although amphiphilic aliphatic polyester smart-blocks are widely used to fabricate injectable self-assembled hydrogels, the degraded byproducts contain carboxylic acid groups that reduce the pH of surrounding environment and may cause damage of encapsulated bioactive molecules and/or induce the inflammation of host tissue. Polypeptides that are enzymatic degradable (Cheng et al., 2012) form zwitterionic amino acid degradation products may overcome this limitation. Many polypeptides (Figure 3), such as poly(L-alanine) (PLAl) (Choi et al., 2008; Kim J. Y. et al., 2009; Park et al., 2011, 2015; Yun et al., 2012; Yeon et al., 2013; Kim et al., 2014; Kye et al., 2014; Patel et al., 2015; Hong et al., 2017; Moon et al., 2017), poly(D,L-alanine) (PDLAl) (Choi et al., 2008, 2010, 2011b; Oh et al., 2008), polyglycine (PG) (Xuan et al., 2016), poly(L-leucine) (PL) (Breedveld et al., 2004; Yang et al., 2009; Song et al., 2012; Zhang et al., 2014, 2015a,b), poly(L-alanine-*co*-L-phenyl alanine) (PLAF) (Jeong et al., 2009; Kim E. H. et al., 2009; Kang et al., 2012a,b; Shinde et al., 2012, 2015; Park et al., 2014; Wei et al., 2017), and poly(γ-alkyl-L-glutamate) (PLG) (Cheng et al., 2012, 2013a,b; Wu et al., 2017; Lv et al., 2018) have been reported to exhibit conformational change upon temperature change that led to the copolymer hydrogel formation in aqueous solution. This section discusses examples of detailed synthesis, gelation mechanisms, and important factors to control the self-assembled gelation of some typical polypeptide temperature-sensitive smart-blocks.

Amphiphilic PLAl and PDLAl smart-blocks can be synthesized via ROP of N-carboxyl anhydride L-alanine and D,L-alanine, respectively, using amino terminated molecules, such as PEG-amine (Choi et al., 2008; Park et al., 2011; Kim et al., 2014). Copolymers of PLAl or PDLAl with PEG, in the form of PEG-PLAl or PEG-PDLAl diblock copolymers, was first introduced in 2008 as peptide-based temperature-induced hydrogels that showed a sol-to-gel phase transition upon increasing temperature (Choi et al., 2008). PLAl underwent transitions from random coils to β-sheets that subsequently become packed β-sheets nanofibers as the polymer concentration increased. The preassembled β-sheet secondary structure of PLAl facilitates the sol-to-gel phase transition, occurred in the physiologically range of 20–40°C, of PEG-PLAl upon increasing temperature due to the further packaging of the β-sheet secondary structure (Figure 2C). However, when D-alanine stereoisomer was introduced, the β-sheet secondary structure transformation of PDLAl only happened at higher copolymer concentration, and therefore, the PEG-PDLAl could only exhibit the gelation at much higher concentration and temperature (>60°C). Switching the arrangement of PDLAl and PLAl blocks in a copolymer molecule also leads to differences in gelation behavior. PEG-PDLAL-PLAL contain PLAl-end-block could form hydrogels at lower concentrations and lower sol-to-gel transition temperature compared to PDLAl-end-block PEG-PLAL-PDLAL hydrogel due to the formation of cylindrical bundles as well as spherical micelles of PLAl-end-block whereas only spherical micelles in PDLAl-end-block copolymer (Park et al., 2011).

Hydrophobicity of peptide smart-blocks is an important and effective tool to regulate the gelation behavior and gel region of their copolymer. Replacing a small portion of L-alanine in PEG-PLAl (Choi et al., 2008) by more hydrophobic L-phenyl-alanine moiety (Park et al., 2014), to form PEG-PLAF copolymers, could reduce critical gel concentration and sol-to-gel transition temperature. A similar trend can also be achieved by increasing the hydrophobicity from methyl to ethyl in γ-alkyl-substituted PLG in PEG-PLG hydrogels while maintaining the same repeat unit of L-glutamate (Cheng et al., 2012). In addition, increasing the length of PLAl (Choi et al., 2008; Yeon et al., 2013; Kim et al., 2014; Kye et al., 2014; Patel et al., 2015) and PLAF (Kang et al., 2012a; Shinde et al., 2012) and PLG (Cheng et al., 2013a, 2018) peptide smart-blocks or number of repeat unit in PLG (Cheng et al., 2012) or decreasing PEG molecular weight (Jeong et al., 2009; Kang et al., 2012a; Cheng et al., 2013a; Yeon et al., 2013; Kye et al., 2014; Hong et al., 2017) could reduce the critical gel concentration or sol-to-gel phase transition temperature. Peptide smart-blocks, such as PLAl (Kim J. Y. et al., 2009; Moon et al., 2017), PDLAl (Oh et al., 2008; Choi et al., 2010, 2011b), PLAF (Kim E. H. et al., 2009; Kang et al., 2012b) and poly(L-alanine-co-L-leucine) (PLAlL) (Moon et al., 2011), were also combined with PPG-contained or natural polymers, e.g., chitosan (Kang et al., 2012b), to prolong retention time, improve mechanical properties, biocompatibility, and degradability of the formed hydrogels for potential biomedical applications. Impressively, PEG-polytyrosine (PEG-Tyr6) could form hydrogel at low concentration (~1.0%) at physiological temperature due to the synergistic capacity of β-sheet conformation and hydrogen bonding of phenolic groups (Huang et al., 2013).

PG with different N-substituted groups was reported to have different responses to temperature change that can be used to fabricate temperature-controlled hydrogels (Xuan et al., 2016). ABC triblock copolymers composed of poly(N-R1 glycine)-poly(N-R2 glycine)-poly(N-R3 glycine), where R is a N-substituted groups, exhibited the sol-to-gel phase transition with increasing temperature that can be adjusted by controlling the polymer solution concentration and the hydrophobic of N-substituted groups. The combination of PL with PLAl, poly(L-lysine) (PK) and poly(glutamic acid) (PE) were also reported to exhibit a sol-gel transition in response to temperature for tissue regeneration (Zhang et al., 2014, 2015a,b). Esterified-PE smart-blocks were also reported to respond to temperature change (Cheng et al., 2012, 2013a,b; Wu et al., 2017; Lv et al., 2018). Changing the block length or γ-substituted group in PE could regulate the gelation of the copolymer hydrogels. For example, increasing the hydrophobicity of γ-substituted groups from methyl or ethyl to n-propyl or butyl significantly decreased the critical gelation temperature (Cheng et al., 2012). In addition, amphiphilic poly(α/β-asparagine) derivatives, synthesized by the reaction of polysuccinimide with a combination of hydrophilic and hydrophobic amines, showed a sharp sol-gel-sol phase transition in an aqueous solution with the ability to alter the gel region via tailoring the side-chain structure of the poly(amino acid)s (Chueh et al., 2007; Takeuchi et al., 2011).

### Poly(Organo-Phosphazene) Smart-Blocks

Poly(organo-phosphazene) (POPP) contains only phosphorous and nitrogen in its backbone that is degradable and easily modified with functional groups, e.g., PEG, L-isoleucine ethyl ester (IleOEt), and carboxylic acid (Figure 3), to fabricate temperature-induced hydrogels for biomedical application. In aqueous solution, these copolymers exhibited a sol-to-gel phase transition with increasing temperature with the gel region covers the physiological temperature. In the POPP smart-blocks, IleOEt group offers the temperature-sensitive property while the remaining carboxylic acid side group (Seo et al., 2017) offer the opportunity for further modification, such as conjugating with anticancer drug (Chun et al., 2009a), cell-adhesive peptide (Chun et al., 2009b), folic acid (a cell-specific targeting moiety; Kim and Song, 2014), and cationic molecules (Park et al., 2010; Kim et al., 2012, 2013; Kim and Song, 2014) to improve the sustained delivery, biocompatibility, and affinity with bioactive molecules.

### Aliphatic Polycarbonate-Based Smart-Blocks

Aliphatic polycarbonate-based smart-blocks (Figure 3) have also attracted the attention from scientists due to its ease of preparation, biocompatibility, low degradation rate, and mechanical properties. PEG-PTMC diblock copolymers exhibited a sol-to-gel-to-sol transition in aqueous solution upon increasing temperature due to the amphiphilic property of PTMC mart-blocks (Kim et al., 2007). The sol-to-gel transition temperature could be controlled by tailoring concentration, molecular weight, and composition of the polymer. However, PTMC smart-block has difficulty in regulating the gelation of its copolymer. The gelation of PTMC-PEG-PTMC triblock copolymers could only be achieved with high molecular weight PEG and the sol-state at low temperature was not observed (Bat et al., 2008; Park et al., 2008). When PCL, a more hydrophobic block, was added to PTMC, the resulted triblock copolymer exhibited a sol-gel-sol phase transition with increase temperature (Park et al., 2008). Multi-block copolymers fabricated by coupling of PTMC with PPG and PEG [(PEG-PPG-PTMC)_x_] via urethane addition reaction exhibited a temperature-induced sol-to-gel phase transition at much lower polymer concentration (<2%) (Loh et al., 2012). Copolymerization of poly(polytetrahydrofuran carbonate) (PTHC) with Pluronic F127 (Loh et al., 2014) or PPG and PEG (Chan et al., 2018) [(PEG-PPG-PEG-PTHC)_x_] or [(PEG-PPG-PTHC)_x_] via urethane reaction were reported for regulating the gel window and hydrogel property with much lower critical gelation concentration.

### Poly(N-Isopropyl Acrylamide) Smart-Blocks

PNIPAAm (Figure 3) is a wildly used temperature-sensitive smart-block for hydrogel preparation. However, there is very limited reports of using PNIPAAm for fabricating injectable temperature-induced physically cross-linked hydrogels for biomedical application due to its low critical transition temperature (~32°C), which is much lower than physiological temperature. PNIPAAm can be combined with other smart-block to regulate the gelation of their copolymers upon heating due to the trigger of hydrogen bond between amide groups. For example, in aqueous solution, copolymer of PNIPAAm with poly(2-hydroxyl ethyl methacrylate) (PHEMA) and poly(methacrylate-PLA) (pMAPLA) in the form of poly(NIPAAm-co-HEMA-co-MAPLA) existed as a sol at low temperature (<10°C) and exhibited a sol-to-gel phase transition when temperature was increased to body condition (Ma et al., 2010). However, the gel shrunk after a day. When PNIPAAm was copolymerized with poly(acrylic acid) (PAAc) and poly(trimethylene carbonate) to form poly(NIPAAm-co-AAc-co-PTMC), the formed hydrogel was stable both *in vitro* and *in vivo* due to the additional crosslinking between PTMC smart-blocks, which enhanced the stability of the formed hydrogel (Fujimoto et al., 2009). Conjugating of PNIPAAm via ATRP of NIPAAm into 2-bromoisobutyryl bromide functionalized un-gellable PCL-PEG-PCL triblock copolymer could trigger the gelation of formed PNIPAAm-PCL-PEG-PCL-PNIPAAm pentablock copolymers (Abandansari et al., 2013). Grafting PNIPAAm into natural polymer is also a potential strategy to fabricate PNIPAAm-based injectable hydrogel. PNIPAAm-grafted chondroitin sulfate underwent a sol-to-gel phase transition with increasing temperature from room temperature to body condition (Lü et al., 2011).

### Other Synthetic Smart-Blocks

Many less popular smart-blocks have been reported to trigger the gelation of their copolymers in aqueous solution upon changing temperature for fabricating injectable hydrogels with highly potential for biomedical applications. These smart-blocks include poly(ethylene/butylene) (Nguyen et al., 2011), poly(propylene fumarate) (Chapanian et al., 2009), polyorthoester (Schacht et al., 2006).

## Self-Assembly Smart-Blocks for Both Temperature- and Ph-Induced Hydrogels

**Figure 4:**
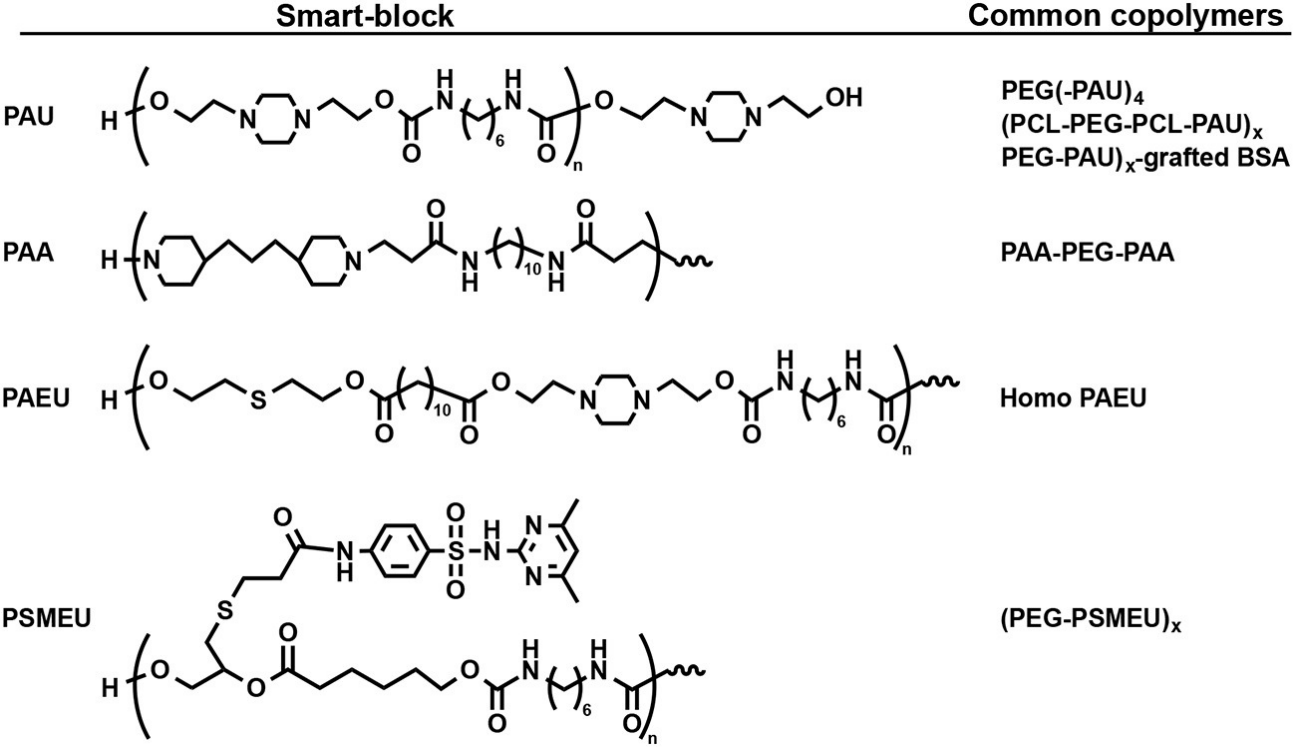
Structure of some smart-blocks for both temperature- and pH-induced self-assemble to fabricate injectable hydrogels.

pH is an important parameter of a living body that may vary between the body fluid, specific organs and disease tissues. Although this work aims to discuss synthetic smart-blocks for temperature-induced gelation, some smart-blocks that are able to trigger a sol-gel phase transition of their copolymers in response to both temperature and pH change for fabrication self-assembled injectable hydrogel are briefly mentioned (Figure 4) (Dayananda et al., 2008; Nguyen et al., 2009; Huynh et al., 2010, 2011b). Temperature and pH-sensitive smart-blocks are polymers with functional groups that can be ionized and de-ionized in response to pH change, including sulfonamide groups and amino group, and also show the self-assemblability in response to temperature. These smart-blocks are soluble at acidic or basic pH (e.g., pH <6.0 or pH > 8.5, respectively) due to the protonation of pH-sensitive moiety and convert to amphiphilic blocks at physiological pH (pH = 7.4) due to the deprotonation (Dayananda et al., 2008; Nguyen et al., 2009; Huynh et al., 2010, 2011b). Their amphiphilic property can further trigger the gelation of their copolymers upon increasing temperature at physiological pH to form a hydrogel.

**Figure 5:**
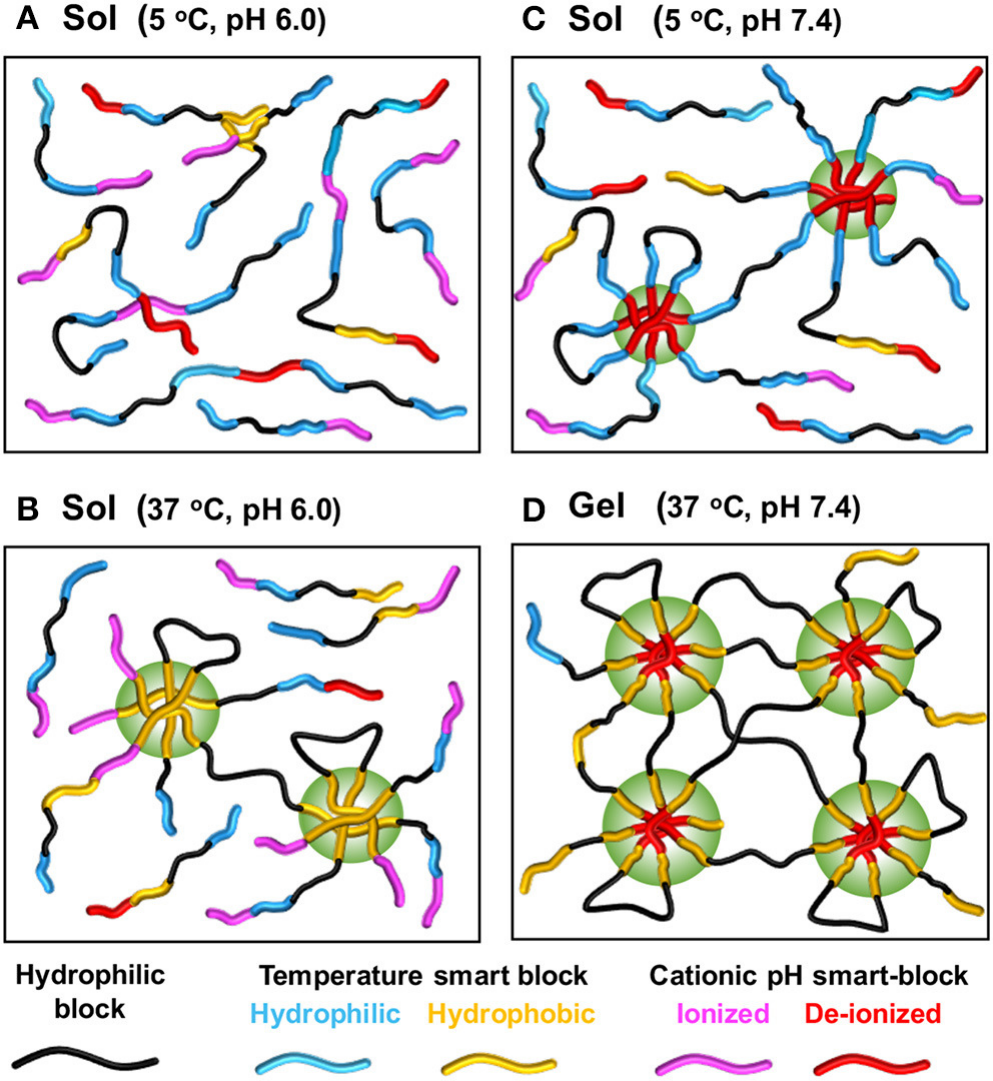
Schematic showing mechanism of sol-gel phase transition upon changing of temperature and pH of temperature- and pH-induced hydrogels. The copolymer contains both temperature-sensitive and pH-sensitive smart-blocks, e.g., (PCL-PEG-PCL-PAU)_x_. Copolymer exists as a solution in aqueous solution at low temperatures and acidic pH **(A)** retains its sol state with solely increasing of temperature **(B)** or pH **(C)** but changes to a gel when both temperature and pH are increasing **(D)**. Generated based on the idea from Dayananda et al. (2008).

There are two main categories of pH-sensitive smart-blocks that can trigger the gelation of their copolymer in different direction. Cationic smart-blocks are amino-contained polymers, including non-degradable PAU (Dayananda et al., 2008; Huynh et al., 2010; Manokruang et al., 2014), poly(amino carbonate urethane) (PACU) (Phan et al., 2016a), poly(amido amine) (PAA) (Nguyen et al., 2009; Nguyen and Lee, 2010) and biodegradable PAEU (Huynh et al., 2011b), which are soluble at acidic pH (e.g., pH <6.0), have amphiphilic property at physiological pH (pH = 7.4) for trigger the gelation upon increasing temperature. For example, cationic PAU smart-blocks, which was synthesized via urethane polyaddition between hydroxyl groups in 1,4-bis(hydroxyethyl) piperazine and isocyanate groups in HDI, is soluble at acidic pH = 6.0 due to the ionization of amino groups. At physiological pH, deionized PAU regulated the sol-to-gel transition of its copolymers, PEG(-PAU)_4_ (Huynh et al., 2010) or (PEG-PAU)_x_-grafted-BSA (Manokruang et al., 2014) or (PCL-PEG-PCL-PAU)_x_ (Dayananda et al., 2008), upon increasing temperature from low (e.g., 5°C) to body temperature (Figure 5). In addition, low molecular weight biodegradable PAEU, synthesized using similar urethane polyaddition reaction, could trigger the its sol-to-gel phase transition upon increasing temperature at physiological pH (Huynh et al., 2011b). In opposite manner, poly(sulfamethazine ester urethane) (PSMEU) anionic smart-blocks, which was synthesized via urethane polyaddition, could trigger the gelation of its (PEG-PSMEU)_x_ multiblock copolymers upon the increasing temperature at physiological pH (Le et al., 2018, 2019).

These temperature and pH-sensitive smart-blocks can also be combined with other mentioned temperature-sensitive smart-blocks to trigger the gelation of their copolymers. For example, copolymers of PAU, PCL and PEG, (PCL-PEG-PCL-PAU)_x_, exhibited the gelation upon increasing pH and/or temperature with the existence of a gel at the physiological condition (Dayananda et al., 2008). In addition, many other pH-sensitive smart-blocks that could not trigger the sol-to-gel phase transition upon increasing temperature at physiological pH but can be combined with other temperature-sensitive smart-bocks to triggered the gelation of their copolymers upon changing pH or increasing the temperature. Those include oligosulfamethazine (OSM) in OSM-PCLA-PEG-PCLA-OSM pentablock copolymers (Shim et al., 2007), and poly(amino ester) (PAE) in PAE-PCL-PEG-PCL-PAE pentablock (Huynh et al., 2008) or (PAE-g-PCL)-PEG-(PAE-g-PCL) triblock copolymers (Zheng et al., 2010). Interestingly, end-capping Pluronic F127 with polyamine could trigger a closed-loop sol-gel-sol phase transition as upon increasing pH and/or temperature (Lee et al., 2009). Amphotectic poly(SM amino urethane) (PSMAU) contains both anionic and cationic moiety in its structure that could trigger the gelation of its copolymer, (PCLA-PEG-PCLA-PSMAU)_x_ multiblock copolymers, to form a closed-loop sol-gel-sol phase transition upon increasing pH and/or temperature due to its solubility at either acidic and basic pH (Huynh et al., 2012).

## Biomedical Application of Temperature-Induced Self-Assembled Hydrogels

Temperature-induced self-assembled hydrogels have been widely used for biomedical application including drug delivery systems, cancer and diabetic therapeutic, wound healing, and tissue regeneration (Kim et al., 2006a, 2014; Huynh et al., 2011a; Chiang et al., 2014; Kye et al., 2014; Nguyen et al., 2015; Patel et al., 2015; Yuan et al., 2015; Chen et al., 2016a,b; Norouzi et al., 2016; Wang S. J. et al., 2016; Khaliq et al., 2017; Liow et al., 2017; Liu et al., 2017; Santovena et al., 2017; Lv et al., 2018; Yu et al., 2018; Cirillo et al., 2019). This section provides a summary of some typical biomedical applications of temperature-induced self-assembled injectable hydrogels (Tables 1, 2).

**Table 1:** Bioactive molecules and animal models for therapeutics.

**Bioactive molecules**	**Smart block**	**Copolymer**	**Animal model**	**References**
**Control cancer**				
Adenoviruses	PSMEU	(PEG-PSMEU)_x_	Mouse	Le et al., 2019
CPT	PLGA	PLGA-PEG-PLGA	Mouse	Yu et al., 2008; Chang et al., 2011
DOX	PCL	PEG-PCL	Mouse	Kang et al., 2011
	PLGA	PLGA(-PEG)_4_	Mouse	Lee et al., 2007
	PPG	(PEG-PPG)_x_	Mouse	Liow et al., 2017
	PPG	PEG-PPG-PEG	Mouse	Khaliq et al., 2017
	PPG/PTHF	(PEG-PPG-PTHF)_x_	Mouse	Chan et al., 2018
DOX + CA4	PLAF	PEG-PLAF	Mouse	Wei et al., 2017
DOX + ^188^Re-Tin colloid	PCL	PCL-PEG-PCL	Mouse	Peng C. L. et al., 2013
DOX + IL-2 + IFN-γ	PLG	PEG-PLG2	Mouse	Lv et al., 2018
DOX + PLK1-shRNA	PLGA	PLGA-PEG-PLGA	Mouse	Ma et al., 2014
DOX + CDDP + MTX	PLGA	PLGA-PEG-PLGA	Mouse	Ma et al., 2015
DTX	PLGA	PLGA-PEG-PLGA	Mouse	Gao et al., 2011
GEM	PCLA	PCLA-PEG-PCLA	Mouse	Phan et al., 2016b
IL-15 +CDDP	PLG	PEG-PLG	Mouse	Wu et al., 2017
IL-2	PLGA	PLGA-PEG-PLGA	Mouse	Samlowski et al., 2006
IRN	PLGA	PLGA-PEG-PLGA	Mouse	Ci et al., 2014
PTX	OSM	OSM-PCLA-PEG-PCLA-OSM	Mouse	Shim et al., 2007
	PCL	PCL-PEG	Mouse	Lee et al., 2010
	PLG	PEG-PLG2	Mouse	Cheng et al., 2013a
	PLGA	PLGA-PEG-PLGA	Rat	Bagley et al., 2007
			Mouse	Zentner et al., 2001
			Pig	Matthes et al., 2007
			Human	Vukelja et al., 2007
	IleOEt	POPP	Mouse	Chun et al., 2009a
	PPG/PCL	(PEG-PPG-PCL)_x_	Mouse	Zheng et al., 2017
	PPG/PHB	(PEG-PPG- PHB)_x_	Mouse	Wu et al., 2016b
	PPG/PLA	(PEG-PPG-PLA)_x_	Mouse	Wu et al., 2016a
PTX + Pt(IV) prodrug	PLGA	PLGA-PEG	Mouse	Shen et al., 2017
rhIL-2	PLA	PEG(-PLLA)_8_ + PEG(-PDLA)_8_	Mouse	Hiemstra et al., 2007
si(Cyclin B1)	IleOEt	POPP	Mouse	Kim et al., 2012
siVEGF	IleOEt	POPP	Mouse	Kim et al., 2013; Kim and Song, 2014
**Control diabetic**				
EXT	PLGA	PLGA-PEG-PLGA	Mouse	Li et al., 2013; Yu et al., 2013
GLP-1	PLGA	PLGA-PEG-PLGA	Rat	Choi et al., 2004
Insulin	PAE	PAE-PCL-PEG-PCL-PAE	Rat	Huynh et al., 2008, 2009
	PLAF	PEG-PLAF	Mouse	Jeong et al., 2009
	PLGA	PLGA-PEG-PLGA	Rat	Kim et al., 2001; Choi and Kim, 2003
Lira	PCGA	PCGA-PEG-PCGA	Mouse	Cheng et al., 2013a; Chen et al., 2016a
**Control pain and others**				
Celecoxib	PCLA	PCLA-PEG-PCLA	Dog Horse Rat	Tellegen et al., 2018 Petit et al., 2015 Petit et al., 2014
pDNA	PCLA	PCLA-PEG-PCLA-grafted BSA	Mouse	Giang Phan et al., 2019
VEGF	PVL	PVL-PEG-PVL	SD rats	Wu et al., 2011

**Table 2:** Bioactive molecules and cell sources for tissue regeneration.

**Bioactive molecules**	**Cells**	**Smart block**	**Copolymer**	**Animal model**	**References**
**Bone regeneration**					
	TMSCs	PLAl	PEG-PLAl	*In vitro*	Kye et al., 2014
BMP-2		PLGA	PLGA-PEG-PLGA	Rat	Santovena et al., 2017
BMP-2, BCP		POPP	POPP	Mouse	Seo et al., 2017
DEX	rBMSCs	PCL	PCL-PE	3D culturing *in vitro*	Kim et al., 2006a
RGD		IleOEt	POPP		Chun et al., 2009b
SIM		PLGA	PLGA-PEG-PLGA	Rat	Yan et al., 2015
**Cartilage regeneration**					
	ADSCs	PG	PG-based triblock	*In vitro*	Xuan et al., 2016
	ADSCs	PLAl	PEG-PLAl	Mouse	Yeon et al., 2013
	BMSCs	PLAl	PEG-P	3D culturing *in vitro*	Park et al., 2015
	BMSCs	PLGA	PLGA-PEG-PLGA		Wang S. J. et al., 2016
	CHONs	PDLAl	PDLAl-PPG-PEG-PPG-PDLAl		Choi et al., 2010, 2011b
	CHONs	PLGA	PEG-PLGA		Peng S. et al., 2013
	CHONs	poly(α/β-asparagine)	poly(α/β-asparagine)		Chueh et al., 2007
	CHONs	PTMC	PTMC-PEG-PTMC		Park et al., 2008
	TMSCs	PLAF	PEG-PLAF		Park et al., 2014
	TMSCs	PLAl	PEG-PLAl		Kye et al., 2014
PRP	SFMSCs	PLGA	PEG-PLGA	Pig	Chiang et al., 2014
**Hepatogenic regeneration**					
HGF	TMSCs	PLAl	PEG-PLAl	3D culturing *in vitro*	Kim et al., 2014
HGF + TUDCA + FGF-4	TMSCs	PLAl	PEG-PLAl		Hong et al., 2017
LB	TMSCs	PLAl	PLAl-PPG-PEG-PPG		Moon et al., 2017
**Neural and muscle regeneration**					
	NSCs	PL	PL-based	Mouse	Zhang L. et al., 2015
	PNIPA	PCL	PCL-PEG	Mouse	Kim M. H. et al., 2010
NGF		PL	PL-based	Mouse	Song et al., 2012
NGF + BDNF	TMSCs	PLAl	PEG-PLAL	*In vitro*	Patel et al., 2015
**Wound healing and scar prevention**					
	Fibroblasts	PLAl	PEG-PLAl	Rat	Yun et al., 2012
5-Fu		PLGA	PLGA-PEG-PLGA	Rat	Yuan et al., 2015
CsA		PLGA	PLGA-PEG-PLGA	Rabbit	Sun et al., 2017
pDNA-polyplex		PSMEU	(PEG-PSMEU)_x_	Mouse	Le et al., 2018

### Temperature-Induced Self-Assembled Hydrogels as Drug Delivery Model Systems

#### Anticancer Drugs Model Systems

A wide range of bioactive molecules has been used as a model drug for testing the release behavior from hydrogel systems. Among those, anticancer drugs are one of the most interested molecular types because hydrogels can be easily injected into the tumor site or deeper sites in the body, which advances their potential application in cancer therapy. These temperature-induced hydrogels exhibited the sustained release of many anticancer drugs, such as doxorubicin (DOX) (Xun et al., 2009; Huynh et al., 2011b; Loh et al., 2012; Manokruang et al., 2014; Guo et al., 2015; Wee et al., 2017; Zhang et al., 2017), paclitacxel (PTX) (Elstad and Fowers, 2009), chlorambucil (Huynh et al., 2010), honokiol (Gong et al., 2009a,b; Gou et al., 2010), and leuprorelin acetate (Rao et al., 2018) with multiple options to adjust the release behavior. The release of the loaded anticancer drugs can be regulated via controlling the property of smart-blocks in the hydrogels. For example, *in vitro* experiments showed that DOX was released from PCLA-PEG-PCLA hydrogels in the course of 2 weeks while further attachment of KRGDKK (Lys-Arg-Gly-Asp-Lys-Lys) peptide at both end of copolymer could increase the sustained release up to 5 weeks (Xun et al., 2009). However, DOX was released in from PLA-PEG-PLA hydrogels in only 1 week (Guo et al., 2015) due to the faster degradation of PLA compared to PCLA. Introducing stimuli-triggered degradation groups into the middle of smart-blocks offers the ability of using stimuli to control the degradation of hydrogels and subsequently the release of loaded bioactive molecules (Zhang et al., 2017). For example, introducing a diselenide linkage at the middle of PCL block in PEG-PCL-PEG copolymers could offer reducing-agent-regulated release of loaded DOX with faster DOX release rate due to hydrogel degradation in the presence of a reducing agent (Zhang et al., 2017). PEG-PCL-PEG hydrogels were also reported to control the release of honokiol over the course of 2–3 weeks (Gong et al., 2009a,b; Gou et al., 2010). Increasing the hydrophobicity of smart-blocks not only triggered the gelation at lower polymer concentrations, but also offered more sustained released of loaded anticancer drugs (Loh et al., 2012; Wee et al., 2017). The hydrogels contain smart-blocks that response to both temperature and pH also exhibited sustained release of DOX (Huynh et al., 2010, 2011b; Manokruang et al., 2014).

#### Proteins in Model Systems

Therapeutic and model proteins, such as insulin (Qiao et al., 2007), human growth hormone (hGH) (Park et al., 2010; Huynh et al., 2012; Shinde et al., 2012, 2015; Phan et al., 2016a) and bovine serum albumin (BSA) (Jeong et al., 1997; Hyun et al., 2007; Moon et al., 2011) are among the molecules served as model drugs for testing the controlled release ability of hydrogel systems. Peptide-based smart-block hydrogels have showed potential in control the release of hGH with sustained release for 1–2 weeks *in vitro* and 4 days in a rat model from PEG-PLAF hydrogels (Shinde et al., 2012, 2015). The improvement in sustained release of hGH was not observed in cationic smart-block contained hydrogels, such as (PEG-PACU)x (Phan et al., 2016a), (PCLA-PEG-PCLA-PSMAU)_x_ (Huynh et al., 2012), protamine-modified POPP (Park et al., 2010). Short release courses of hGH from all these hydrogels was attributed to its naturally hydrophilicity. In addition, hydrogels with faster degradation rate offered shorter controlled release course while hydrogels contained peptide block could offer more sustained protein release due to the addition of hydrogen bonds between protein and peptide segments in the hydrogels. For example, BSA was released from PEG-PLLA hydrogel in 10 day (Jeong et al., 1997) while it was sustained for 3 and 4 weeks from PEG-PCL (Hyun et al., 2007) and PLAlL-PPG-PEG-PPG-PLAlL (Moon et al., 2011) hydrogels, respectively.

#### Other Molecules in Model Systems

Many other drugs and model molecules have also been used to confirm the potential application of a hydrogel system as a drug delivery system. PLGA-PEG-PLGA hydrogels could provide the sustained delivery of dexamethasone (DEX) and DEX acetate in ocular environment (Gao et al., 2010; Zhang L. et al., 2015). The release of anti-infection molecules, such as lysozyme (Sim et al., 2015), natamycin (Loh et al., 2014), and rifampicin (Jiang et al., 2007) were also performed from PCLA-PEG-PCLA (Sim et al., 2015), (PEG-PPG-PEG-PTHF)_x_ (Loh et al., 2014), and PCGA-PEG-PCGA hydrogels (Jiang et al., 2007), respectively. PCLA-PEG-PCLA hydrogels also showed potential in vaccine delivery (Wang X. et al., 2016) while PEG-PLGA-PEG (Jeong et al., 2000) and Pluronic (Jung et al., 2017) hydrogels showed the ability in sustained delivery of pDNA and pain control molecules, respectively.

### Controlled Release of Bioactive Molecules for Therapeutics

#### Cancer Therapeutics

There are enormous number of studies that reported the used of smart-block based temperature-induced self-assembled hydrogels for delivery of anticancer drugs and other bioactive molecules for controlling the tumor growth or clearance tumor in animal models (Table 1). Many designed systems with tunable release behavior and high bioactive efficacy have been developed. PPG-based hydrogels exhibited the sustained release of DOX (Khaliq et al., 2017; Liow et al., 2017; Chan et al., 2018) and PTX (Wu et al., 2016a,b; Zheng et al., 2017) for suppression tumor growth from 1 to 4 weeks. The release of anticancer drugs and resultant tumor suppressing ability could be controlled via changing the designed structure of polymer hydrogels. For example, PTX-loaded (PEG-PPG-PHB)_x_ hydrogels (Wu et al., 2016b) could suppress the tumor growth for 4 weeks while PTX-loaded (PEG-PPG-PLA)_x_ hydrogels (Wu et al., 2016a) were reported to show the control tumor growth in 1 week due to the faster degradation of PLA smart-blocks.

**Figure 6:**
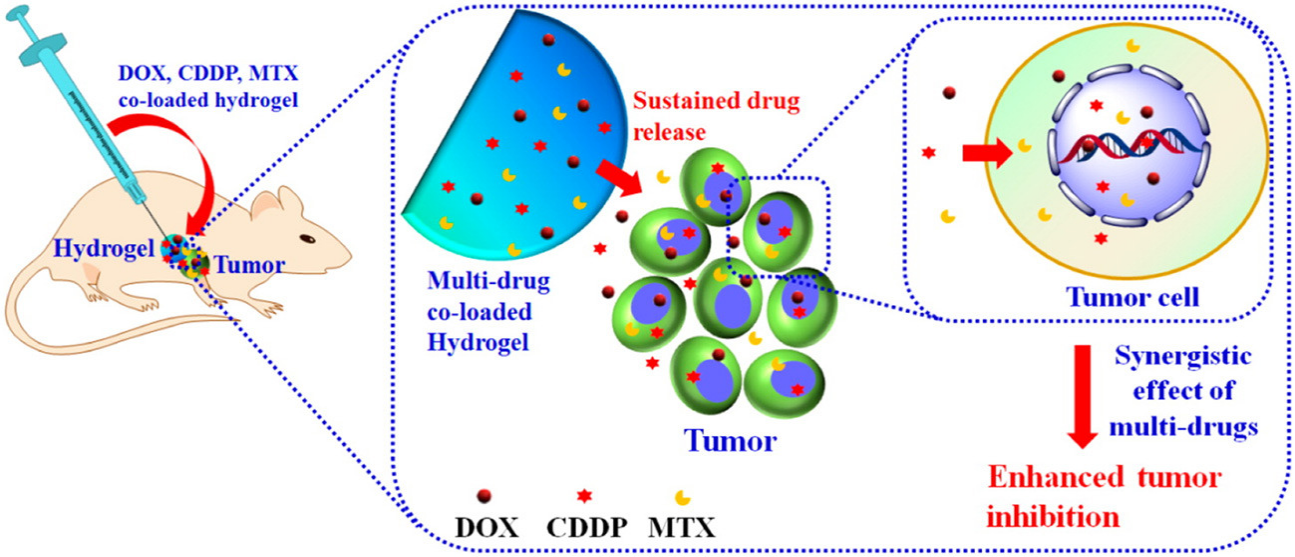
Schematic illustration the localized, sustained co-delivery of multiple anticancer drugs (DOX, CDDP, and MTX) from injectable hydrogels for obtaining the synergistic tumor suppression. Reproduced from Ma et al. (2015) with permission from American Chemical Society.

**Figure 7:**
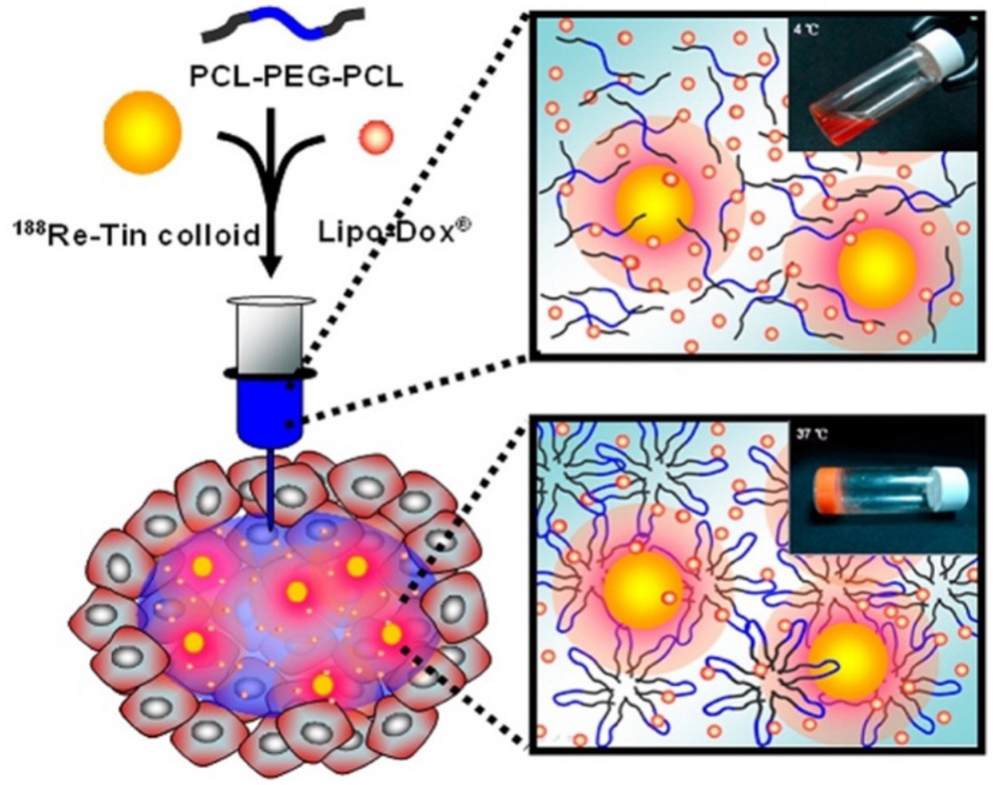
Schematic represents a PCL-PEG-PCL solution loaded with Lipo-DOX and ^188^Re-Tin colloid at 4°C before injecting and a formed hydrogel at 37°C or after being injected to the tumor site for control tumor growth. Reproduced from (Peng C. L. et al., 2013) with permission from American Chemical Society.

Many aliphatic polyester smart-blocks hydrogels have been used for delivery of anticancer drugs and other bioactive molecules to control tumor growth in animal models (Zentner et al., 2001; Samlowski et al., 2006; Bagley et al., 2007; Matthes et al., 2007; Yu et al., 2008; Chang et al., 2011; Gao et al., 2011; Ci et al., 2014; Ma et al., 2014, 2015; Phan et al., 2016b) and even in human patients (Vukelja et al., 2007). Among these, PLGA-based hydrogels are the most popular for cancer therapy. PCLA-PEG-PCLA hydrogels were reported to deliver anticancer drugs, such as gemcitabine (GEM) (Phan et al., 2016b), irinotecan (IRN) (Ci et al., 2014), camptothecin (CPT) (Yu et al., 2008; Chang et al., 2011), docetaxel (DTX) (Gao et al., 2011), cisplatin (CDDP) and methotraxate (MTX) (Ma et al., 2015), DOX (Ma et al., 2014, 2015), and PTX (Zentner et al., 2001; Bagley et al., 2007; Matthes et al., 2007; Vukelja et al., 2007), immunotherapy agent, e.g., interleukin-2 (IL-2) (Samlowski et al., 2006), and RNA molecule, e.g., polo-like kinase 1 shRNA (PLK1-shRNA) (Ma et al., 2014) for controlling tumor growth (Table 1). For example, GEM was sustained release from montmorillonite-functionalized PCLA-PEG-PCLA hydrogels for more than 1 week *in vitro* and could suppressed tumor growth in pancreatic tumor-bearing mice for more than 6 weeks (Phan et al., 2016b). Co-delivery of DOX, CDDP, and MTX from PCLA-PEG-PCLA hydrogels could increase efficacy in suppressing tumor growth in tumor-bearing nude mice compared to groups treated with single or dual drugs (Figure 6) (Ma et al., 2015). Co-delivery of PLK1-shRNA and DOX was also reported to provide the synergistic effect in treatment of cancer in nude mice due to the antitumor effect of DOX and ability of PLK1-shRNA to silence PLK1 expression for higher apoptosis effect (Ma et al., 2014). Other PLGA-based hydrogel, e.g., PLGA(-PEG)_4_ (Lee et al., 2007), PEG-PLGA (Shen et al., 2017), and aliphatic ester smart-block based hydrogels, e.g., PEG-PCL (Lee et al., 2010; Kang et al., 2011) and PCL-PEG-PCL (Peng C. L. et al., 2013) also showed promise in control the release of anticancer drugs for suppressing tumor growth. Importantly, encapsulation of therapeutic radionuclide (^188^Re-Tin colloid) into PCL-PEG-PCL (Peng C. L. et al., 2013) could inhibit the tumor growth for 32 days. The antitumor effect was significantly enhanced when DOX and ^188^Re-Tin colloid were co-delivered, which showed the tumor disappear in 75% animal after 31 days treatment (Peng C. L. et al., 2013). Figure 7 is reproduced from ref (Peng C. L. et al., 2013) showing a schematic represent a copolymer solution loaded with Lipo-DOX and ^188^Re-Tin colloid at 4°C in a syringe that forms a gel at 37°C or after being injected to the tumor site for control tumor growth.

Peptide smart-block hydrogels exhibited excellent ability in controlling the release of bioactive molecules, including anticancer drugs (Cheng et al., 2013a; Wei et al., 2017; Wu et al., 2017; Lv et al., 2018), and immunotherapy agent (Wu et al., 2017; Lv et al., 2018) for subduing tumor growth in animal models. PTX loaded PLG-PEG-PLG hydrogels could sustained the release of PTX and efficiently suppressed the tumor growth in BALB/c nude mice for up to 21 days (Cheng et al., 2013a). Co-delivery of anticancer drugs and immunotherapy agents from peptide-based hydrogels was reported to provide synergistic effect in subduing tumor growth that has significantly higher efficacy compared to the delivery of single molecule (Wei et al., 2017; Wu et al., 2017; Lv et al., 2018). For example, co-delivery of DOX and combretastatin A4 (CA4) from PEG-PLAF hydrogels could inhibit the tumor growth in BALB/c mouse for up to 28 days with much smaller tumor volume compared to single molecule treatment (Wei et al., 2017).

**Figure 8:**
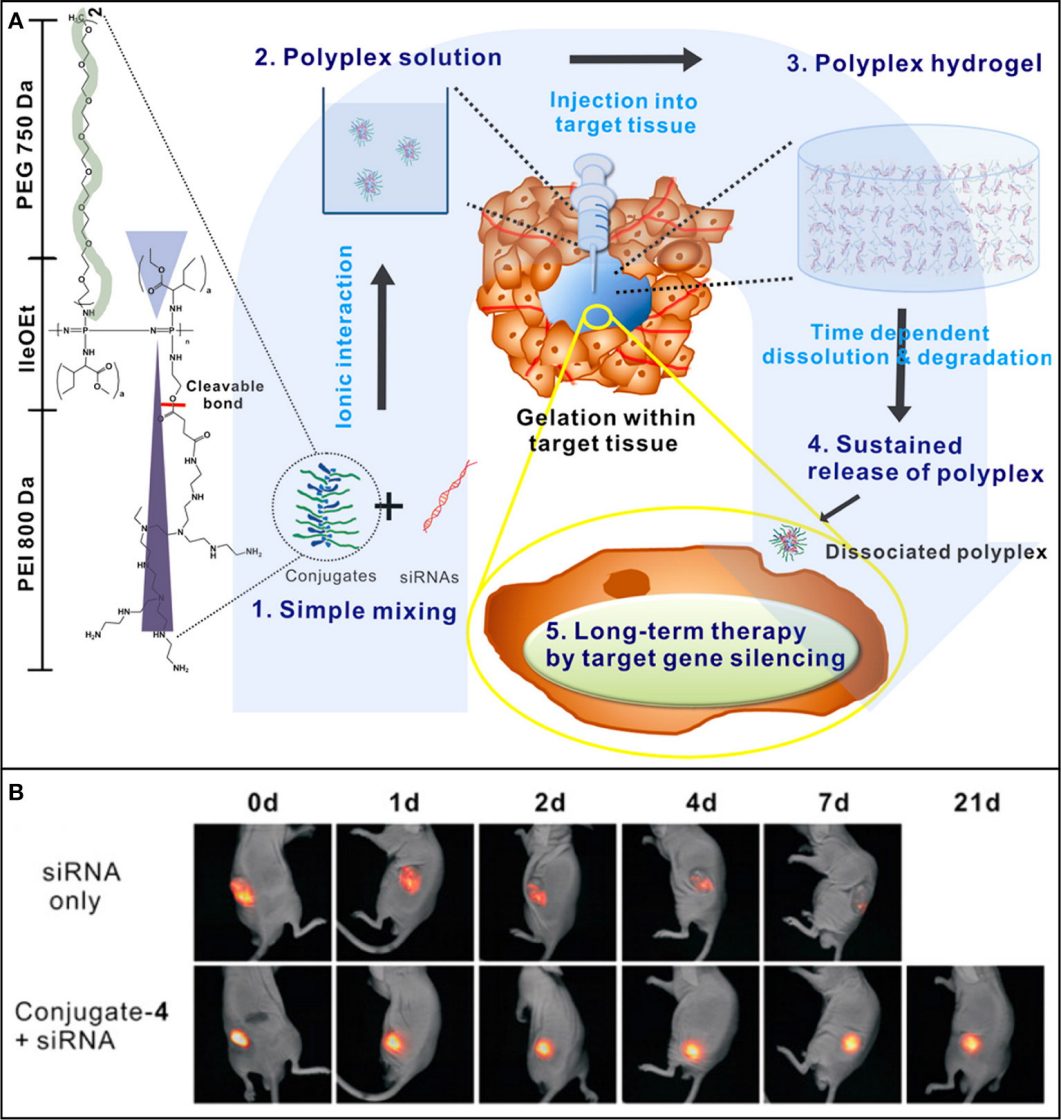
**(A)** Schematic showing the concept of PEI-conjugated POPP injectable hydrogel for localized and long-term delivery of siRNA. By simply mixing (1), the polyplexes (2) form by ionic interactions between conjugated PEI and siRNAs, and their aqueous solution transforms to a gel (3) after being injected into tumor site for sustained siRNA delivery (4) and long-term RNAi therapy via target gene silencing. **(B)** *In vivo* sustained siRNA retention of fluorescent cy5.5-tagged si(Cyclin B1) at tumor site after injection of siRNA-loaded hydrogel. Reproduced from Kim et al. (2012) with permission from American Chemical Society.

POPP possess abundant pendent functional groups that can be easily modified to improve the retention of loaded bioactive molecules. POPP modified with protamine (Kim et al., 2013) or PEI (Kim et al., 2012; Kim and Song, 2014) could provide the sustained delivery of siRNA against vascular endothelial growth factor (siVEGF) (Kim et al., 2013; Kim and Song, 2014) or siRNA against cyclin B1(siCyclin B1) (Kim et al., 2012) for suppressing the tumor growth in mice. For example, siVEGF loaded in protamine-conjugated POPP was sustained released and limited the growth of tumor for 4 weeks (Kim et al., 2013). PEI-conjugated POPP can form cationic interaction with RNA to provide the sustained presentation of siRNA at tumor site (Figure 8) to enhance the long-term RNAi-mediated tumor inhibition via target gene silencing (Kim et al., 2012). When protamine was replaced by PEI and folic acid, a cancer cell targeted molecule, the siVEGF loaded hydrogels could completely inhibited the tumor growth Balb/c nude mice for 30 days (Kim and Song, 2014). In addition, POPP was also able to sustain the delivery of chemically conjugated PTX that suppressed tumor growth for more than 30 days (Chun et al., 2009a).

#### Diabetic Treatment

Smart-block hydrogels have been widely used for delivery bioactive molecules, such as insulin (Kim et al., 2001; Choi and Kim, 2003; Qiao et al., 2007; Huynh et al., 2008, 2009), liraglutide (Lira) (Chen et al., 2016a,b), exenatide (EXT) (Li et al., 2013; Yu et al., 2013), and incretin hormone glucagon-like peptide-1 (GLP-1) (Choi et al., 2004), to control glucose level in diabetic animal models. PLGA-PEG-PLGA hydrogels were reported to provide the sustained delivery of insulin over the course of 2 weeks *in vitro* and 10–15 days in rat models (Kim et al., 2001; Choi and Kim, 2003; Qiao et al., 2007). Peptide-based smart-block hydrogels (PEG-PLAF) offered longer insulin sustained release with 18 days *in vitro*, and an injection of insulin-loaded hydrogel could reduce glucose level in diabetic mice for 18 days (Jeong et al., 2009). However, glucose level in treatment group was higher than that in normal mice from day 4. Adding cationic moiety to the hydrogel could prolong the sustained release of insulin both *in vitro* and *in vivo*. Insulin was released from PAE-PCL-PEG-PCL-PAE hydrogels in 30 days *in vitro* and ~20 days *in vivo* that maintained low blood glucose in diabetic rats for more than 12 days (Huynh et al., 2008, 2009).

PLGA-PEG-PLGA hydrogels also sustained the *in vitro* release of EXT for 1 week and the subcutaneous injected EXT-loaded hydrogels could maintain blood glucose level of type-II diabetic db/db and ICR mice in the normal range for up to 7 days (Li et al., 2013; Yu et al., 2013). Lira, an antidiabetic polypeptide, was sustained released from PLGA-PEG-PLGA hydrogels for more than 10 days (Chen et al., 2016a,b). A single injection of Lira-loaded hydrogels in diabetic db/db mice showed hypoglycemic efficacy for up to 1 week. In addition, the sustained release of GLP-1 from PLGA-PEG-PLGA hydrogels more than 10 days after single subcutaneous injection in to diabetic rats that led to stimulation of insulin secretion to improve glucose tolerance for more than 1 week (Choi et al., 2004).

#### Chronic Pain Relief

PLGA-PEG-PLGA hydrogels also showed great potential in locally sustained drug delivery for chronic pain control (Petit et al., 2014, 2015; Tellegen et al., 2018). Celecoxib was sustained released from the hydrogels for more than 3 months *in vitro* and more than 8 weeks in knee joints of healthy Wistar rats without cytotoxicity after a single injection of celecoxib-loaded hydrogels (Petit et al., 2014). Locally sustained release of celecoxib for 4 weeks was also observed with a single injection of celecoxib-loaded hydrogels into the right middle carpal joint healthy horses (Petit et al., 2015). The formulation was also reported as a safe and effective approach to control back pain in dog with significantly pain reduction for up to 6 months (Tellegen et al., 2018).

#### Other Therapeutics

VEGF-conjugated PVL-PEG-PVL hydrogels showed potential for myocardial and functional recovery stabilizing myocardial infarct and inducing angiogenesis in myocardial infarction SD rats (Wu et al., 2011). PCLA-PEG-PCLA grafted BSA hydrogels provide the sustained release of polyplex-pDNA for more than 10 days in mice. The release polyplex-pDNA activated a robust antigen-specific immune response, which might be potential for the formulation of vaccine against Alzheimer's disease (Giang Phan et al., 2019).

### Temperature-Induced Self-Assembled Hydrogels for Tissue Regeneration

#### Bone Regeneration

Although injectable self-assembled hydrogels possess low mechanical property that may limits their potential application in bone regeneration, some hydrogel systems have been reported to deliver bioactive molecules and/or cells to enhance the regeneration of bone (Table 2). For example, PEG-PCL and RGD-conjugated POPP hydrogels were reported as injectable scaffolds for delivery of rat bone marrow stromal cells (rBMSCs) and DEX (Kim et al., 2006a), and rabbit MSCs (Chun et al., 2009b) to enhance the ectopic bone formation. Delivery of simvastatin (SIM) from PCLA-PEG-PCLA hydrogels to femur bone defects of Wistar rats were reported to significant enhance bone formation (Yan et al., 2015). PCLA-PEG-PCLA hydrogels could also sustain the delivery of bone morphogenetic protein 2 (BMP-2) in SD rats for 9 days (Santovena et al., 2017). Loading bare polystyrene microspheres (MSs) or MSs functionalized with phosphate or carboxylate group into PEG-PLAL hydrogels could guide the osteogenesis of co-encapsulated tonsil-tissue-derived mesenchymal stem cells (TMSCs) (Kye et al., 2014). Importantly, POPP modified with -COOH side-groups were reported to provide the sustained release of BMP-2 for 1–3 months. Injection BMP-2-loaded POPP hydrogels with/with-out biphasic calcium phosphate ceramic (BCP) submicron particles into 5 mm critical-sized bone defects in C57BL/6 mice could significantly enhance the healing rate (Seo et al., 2017).

#### Cartilage Regeneration

Temperature-induced self-assembled hydrogels have showed their potential in cartilage regeneration by supporting the 3D culture of chondrocytes (CHONs) or enhancing the chrondrogenesis of encapsulated MSCs (Table 2). BMSCs encapsulated in PLGA-PEG-PLGA hydrogels underwent the chondrogenic differentiation *in vitro*, demonstrated by upregulating expression of chondrogenic markers and increasing glycosaminoglycan (GAG) content (Wang S. J. et al., 2016). PEG-PLGA hydrogels were also reported as platforms for CHON 3D culture with high cell viability and strong chondrogenic activity (Peng S. et al., 2013) or supporting chondrogenesis of encapsulated synovial fluid mesenchymal stem cells (SFMSCs) (Chiang et al., 2014). PEG-PLGA hydrogels encapsulated with SFMSCs and platelet-rich plasma (PRP) could induce the chondrogenesis of SFMSCs and increase cell growth and maturation of CHONs to significantly enhance the cartilage focal defects in pig model (Chiang et al., 2014).

Peptide smart-block based hydrogels were reported as good candidates for cartilage regeneration. TMSCs were reported to preferentially undergo chondrogenesis with high expressions of type II collagen and sulfated glycosaminoglycan when being encapsulated in PEG-PLAF hydrogels and cultured in induction media supplemented with adipogenic, osteogenic, or chondrogenic factors (Park et al., 2014). PEG-PLAl hydrogels exhibited the capability to regulate the chondrogenesis of encapsulated adipose-tissue-derived stem cells (ADSCs) (Yeon et al., 2013), BMSCs (Park et al., 2015), and TMSCs (Kye et al., 2014). Subcutaneous injection PEG-PLAl aqueous solution loaded with BMSCs (Park et al., 2015) or ADSCs (Yeon et al., 2013) in mice led to the chondrogenesis of encapsulated cells with excellent expressions of early chondrogenic biomarkers. PDLAl-PPG-PEG-PPG-PDLAl (Choi et al., 2010, 2011b) and poly(α/β-asparagine)-based (Chueh et al., 2007) hydrogels can also be used as platforms for CHON 3D culture *in vitro* with high cell viability and strong chondrogenic activity. PG-based ABC triblock copolymer hydrogels could induce chondrogenesis of encapsulated human ADSCs demonstrated by upregulating the expressing of chondrogenic marker (Xuan et al., 2016).

#### Neuron Regeneration

Peptide smart-block based hydrogels possess same stiffness with brain tissue that have been injected into mouse forebrain with minimal detectable toxicity in the central nervous system (CNS) and good integration with brain tissue (Yang et al., 2009). The PL-based hydrogels served as depots for localized and sustained release of bioactive nerve growth factor (NGF) in the CNS that maintained hypertrophy of local forebrain cholinergic neurons for at least 4 weeks (Song et al., 2012). The hydrogels were also reported to deliver hydrophobic molecules that alter gene expression of CNS cells in a locally restricted area in the forebrain (Zhang et al., 2014), or to deliver neural stem cells (NSCs) into an injured CNS (Zhang et al., 2015b). Delivered NSC distributed in non-neural lesion cores and integrated with surrounding healthy neural cells to supported regenerate the host nerve fibers (Zhang et al., 2015b). PEG-PLAL hydrogels encapsulated with NGF and brain derived neurotropic growth factor (BDNF) loaded alginate MSs could sustained the release growth factors from 12 to 18 days (Patel et al., 2015). Cultured TMSCs in growth factors loaded hydrogels exhibited shape change from spherical shape to multipolar elongation with significantly higher expressions of the neuronal biomarkers. A summary is presented in Table 2.

#### Liver Regeneration

Peptide smart-block based hydrogels showed their potential in liver regeneration due to their similar stiffness compared to liver tissue (Table 2). Encapsulation of hepatogenic growth factors (HGF) alone (Kim et al., 2014) or in combination with tauroursodeoxycholic acid (TUDCA) and fibroblast growth factor 4 (FGF-4) (Hong et al., 2017) could provide the sustain delivery of growth factors for guiding the hepatogenic differentiation of co-encapsulated TMSCs, demonstrated by expression of hepatogenic biomarker and hepatocyte-specific biofunctions. Conjugation of lactobionic acid (LB) to PLAl-PPG-PEG-PPG hydrogels could also improve the hepatogenic differentiation of the encapsulated TMSCs (Moon et al., 2017).

#### Wound Healing and Scar Prevention

Hydrogels are good platforms to deliver bioactive molecules and/or cells to improve the wound healing rate (Table 2). PEG-PLAl hydrogels have been reported for encapsulation of fibroblasts to improve the wound healing on incisions of rat skin with significantly better wound closure and skin tissue regeneration compared to groups treated with cell-free gels or PBS (Yun et al., 2012). Release of polyplex-DNA loaded in the (PEG-PSMEU)_x_ hydrogels accelerated the wound healing process in mice, demonstrated by effectively sealing the ruptured skin, absorbing wound exudates, and promoting the tissue regeneration in the wounded area (Le et al., 2018). Bare PCLA-PEG-PCLA-grafted gelatin hydrogels could not only adhere on skin tissues and effectively sealed the wounds, accelerated cutaneous wound healing, and promoted tissue regeneration in the wound area (Turabee et al., 2019).

**Figure 9:**
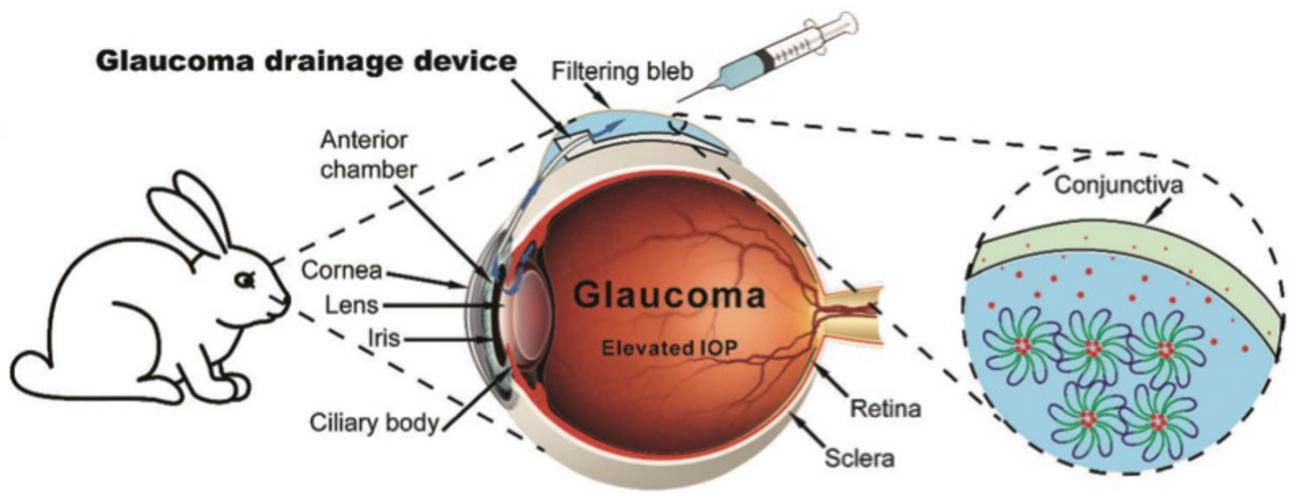
Schematic showing the operated-installation of glaucoma drainage device (GDD) followed by subconjunctival injection of drug-loaded hydrogels. The post-operative scarring was significantly inhibited due to the sustained release of drug from the hydrogel. The function of the filtering bleb was effectively maintained, enabling open drainage of aqueous humor through the GDD tube. Reproduced from Sun et al. (2017) with permission from The Royal Society of Chemistry.

Preventing scar and post-operative abdominal adhesions is very important in medical operation. Cyclosporine A (CsA), an anti-fibrotic drug to inhibit post-operative scarring, was sustained release from PLGA-PEG-PLGA hydrogels for over 2 months *in vitro*. The sustained delivery of CsA from hydrogels inhibited post-surgical scar formation and promoted bleb survival in rabbits after glaucoma filtration surgery (Figure 9) (Sun et al., 2017). PLGA-PEG-PLGA hydrogels also sustained the release of 5-fluorouracil (5-Fu) to prevent post-operative abdominal adhesions in sutured Achilles tendon model of rats (Yuan et al., 2015). Bare hydrogels based on PLGA, PCLA and PLA were also reported to reduce the post-operative abdominal adhesions in animal models (Zhang et al., 2011; Yu et al., 2014a; Shi et al., 2016; Lei et al., 2017; Li et al., 2017).

## Conclusion and Future Outlook of Self-Assembly Smart-Block Based Temperature-Induced Injectable Hydrogels

A summary in development of self-assembly smart-block for fabrication of temperature-induced injectable physically cross-linked hydrogels and their potential biomedical applications have been conducted. The development and achievement in biomedical applications of these smart-block based hydrogels have showed remarkable progress in over the last 20 years. These injectable hydrogels offer many advantages such as cytocompatibility, non-invasive administration, tunable mechanical properties, highly permeability, controllable degradability, injectability, and capacity to deliver bioactive molecules and/or cells for a wide range of biomedical applications. These hydrogel systems have confirmed their potential application as drug delivery systems, cancer and disease therapeutics, and delivery of bioactive molecules and cells for tissue regeneration.

Although these self-assembled injectable hydrogels have offered many advantages and potential, several important challenges still remain and need to be carefully considered in designing the future approaches. The challenges include:

- First, the majority of biodegradable hydrogels are ester based polymers with carboxylic acid bearing degraded byproducts, which might cause a decreasing of local pH and/or inflammation to the surrounding tissues, and damage of loaded bioactive molecules.
- Second, the hydrogel degradation rate is an important factor to control the release of therapeutic molecules; therefore, the balance between hydrogel bulk degradation and surface degradation should be considered for sustaining the delivery of these molecules.
- Third, controlling the initial burst release of loaded bioactive molecules, especially for proteins, growth factors and RNA molecules, from these delivery systems is crucial as it may reduce the serving period of these carrier systems. Loading bioactive molecules into nanoparticles (NPs) or MSs followed by encapsulation of these NPs/MSs in the hydrogels (Giang Phan et al., 2019) or directly tethering them to the network (Nguyen et al., 2019) for prolong the sustained delivery should be put in mind.
- Fourth, several reports have proved that the combination of multiple drugs and/or molecules can enhance effects in disease or cancer therapeutics (Peng C. L. et al., 2013; Ma et al., 2014; Wei et al., 2017; Lv et al., 2018); therefore, this direction should be further investigated to maximize their synergistic therapeutic effect with minimal side effects.
- Fifth, the difference between animal models and human patients is the most difficult challenge that cause a huge delay in potential clinicalization and commercializing of developed approach that may have significantly impact in improving human health.

In summary, clearly understanding the polymer structure-property relationship should allow designing of self-assembly smart-blocks and their hydrogel systems with desired performance. Important properties of smart-blocks include assembability, degradability, property of their degraded byproducts, mechanical property of their formed hydrogels, gelation time, interaction between smart-blocks with bioactive molecules (proteins, DNAs, RNAs), and ability to covalently conjugate bioactive molecules to the network. In addition, the nature property and availability of bioactive molecules, and the availability of cell sources and developed technologies are among important factors to achieve an ideal hydrogel system. Furthermore, pre-clinical pilot animal studies need to be moving forward for developing clinical trial approaches to further enhance human health.

## Author Contributions

TH, LS, DN, DH, and CH summarized the information and prepared the manuscript. CH supervised the whole process.

### Conflict of Interest

The authors declare that the research was conducted in the absence of any commercial or financial relationships that could be construed as a potential conflict of interest.
